# Assessment of a complete and classified platelet proteome from genome-wide transcripts of human platelets and megakaryocytes covering platelet functions

**DOI:** 10.1038/s41598-021-91661-x

**Published:** 2021-06-11

**Authors:** Jingnan Huang, Frauke Swieringa, Fiorella A. Solari, Isabella Provenzale, Luigi Grassi, Ilaria De Simone, Constance C. F. M. J. Baaten, Rachel Cavill, Albert Sickmann, Mattia Frontini, Johan W. M. Heemskerk

**Affiliations:** 1 Department of Biochemistry, CARIM, Maastricht University, P.O. Box 616, 6200 MD Maastricht, The Netherlands; 2 Leibniz-Institut Für Analytische Wissenschaften-ISAS-E.V, Dortmund, Germany; 3 Department of Haematology, University of Cambridge, National Health Service Blood and Transplant (NHSBT), Cambridge Biomedical Campus, Cambridge, UK; 4 Institute for Molecular Cardiovascular Research (IMCAR), University Hospital RWTH, Aachen, Germany; 5 Department of Data Science and Knowledge Engineering, FSE, Maastricht University, Maastricht, The Netherlands; 6 Medizinische Fakultät, Medizinische Proteom-Center, Ruhr-Universität Bochum, Germany; 7 Department of Chemistry, College of Physical Sciences, University of Aberdeen, Aberdeen, UK; 8 Institute of Biomedical & Clinical Science, College of Medicine and Health, University of Exeter Medical School, Exeter, UK

**Keywords:** Cardiovascular biology, Cardiovascular diseases, Haematological diseases, Cell biology, Molecular medicine

## Abstract

Novel platelet and megakaryocyte transcriptome analysis allows prediction of the full or theoretical proteome of a representative human platelet. Here, we integrated the established platelet proteomes from six cohorts of healthy subjects, encompassing 5.2 k proteins, with two novel genome-wide transcriptomes (57.8 k mRNAs). For 14.8 k protein-coding transcripts, we assigned the proteins to 21 UniProt-based classes, based on their preferential intracellular localization and presumed function. This classified transcriptome-proteome profile of platelets revealed: (i) Absence of 37.2 k genome-wide transcripts. (ii) High quantitative similarity of platelet and megakaryocyte transcriptomes (R = 0.75) for 14.8 k protein-coding genes, but not for 3.8 k RNA genes or 1.9 k pseudogenes (R = 0.43–0.54), suggesting redistribution of mRNAs upon platelet shedding from megakaryocytes. (iii) Copy numbers of 3.5 k proteins that were restricted in size by the corresponding transcript levels (iv) Near complete coverage of identified proteins in the relevant transcriptome (log2fpkm > 0.20) except for plasma-derived secretory proteins, pointing to adhesion and uptake of such proteins. (v) Underrepresentation in the identified proteome of nuclear-related, membrane and signaling proteins, as well proteins with low-level transcripts. We then constructed a prediction model, based on protein function, transcript level and (peri)nuclear localization, and calculated the achievable proteome at ~ 10 k proteins. Model validation identified 1.0 k additional proteins in the predicted classes. Network and database analysis revealed the presence of 2.4 k proteins with a possible role in thrombosis and hemostasis, and 138 proteins linked to platelet-related disorders. This genome-wide platelet transcriptome and (non)identified proteome database thus provides a scaffold for discovering the roles of unknown platelet proteins in health and disease.

## Introduction

Platelets are generated in the bone marrow as cell fragments from hematopoietic stem cells that are differentiated into megakaryocytes. In the circulating, the mature platelets control many blood-related processes both in health and disease. These functions extend from blood vessel-lymph separation and maintenance of vascular integrity to allowing hemostasis, promoting arterial thrombosis, regulating inflammatory, immune and infection processes; and even facilitating tumor progression^1,2^. The ultrastructure and the protein/RNA composition of a platelet, determined during their ontogenesis, allows the execution of all these functions. However, comparative studies of the molecular composition and structure of platelets in relation to their functions and megakaryocytic origin are still missing.

Although platelets do not contain a nucleus, they are equipped with mitochondria, several types of storage granules and multiple intracellular membrane structures, including endoplasmic reticulum (smooth and rough), a likely rudimentary Golgi apparatus, lysosomes, peroxisomes and endosomes^3–5^. Characteristic large invaginations, designated as open canicular or dense tubular system, make up ~ 1% or the cell volume and are filled with blood plasma components. A well-developed actin-myosin and tubulin cytoskeleton is required for proplatelet formation, micro- organization of the membrane structures, and mediates activation-dependent structural changes^6–9^. Whether the full repertoire of metabolic enzymes is present in platelets is still unclear, while the glucose metabolism is well-developed^10,11^. Furthermore, the ribosomal mRNA translation machinery is retained as well as elements of protein processing and trafficking and a repertoire of proteolytic processes in the proteasome^12,13^. Overviews point to a battery of receptors and channels, multiple adaptor molecules and small molecule GTP-binding proteins (G-proteins), and large protein kinase and phosphatase networks^2,14^.

Human genetic studies supported by mouse models show that hundreds and possibly thousands of platelet-expressed proteins contribute to thrombosis and hemostasis^15^. We reasoned that assembling the complete (quantitative) proteome and transcriptome of human platelets can provide a much better understanding of the molecules that determine platelet structure and functions in health and disease. As earlier platelet proteomes, reported in single articles, are limited in the numbers of identified proteins^16–18^, there is a need to integrate multiple proteomic studies based on the same methodology. While the number of genes detected in available transcriptomes of platelets and megakaryocytes are a magnitude higher^19–21^, these do not extend to the whole genome. Here, we combined multiple proteomes with the genome-wide RNA database of platelets and megakaryocytes generated by the Blueprint consortium^22,23^, and integrated these into a platelet structure and function- based protein classification system, for defining the full platelet proteome. Detailed analysis of this database provided novel insights into the structure–function relations of platelets.

## Results

### Function-based classification of platelet proteins in merged proteome

**Figure 1:**
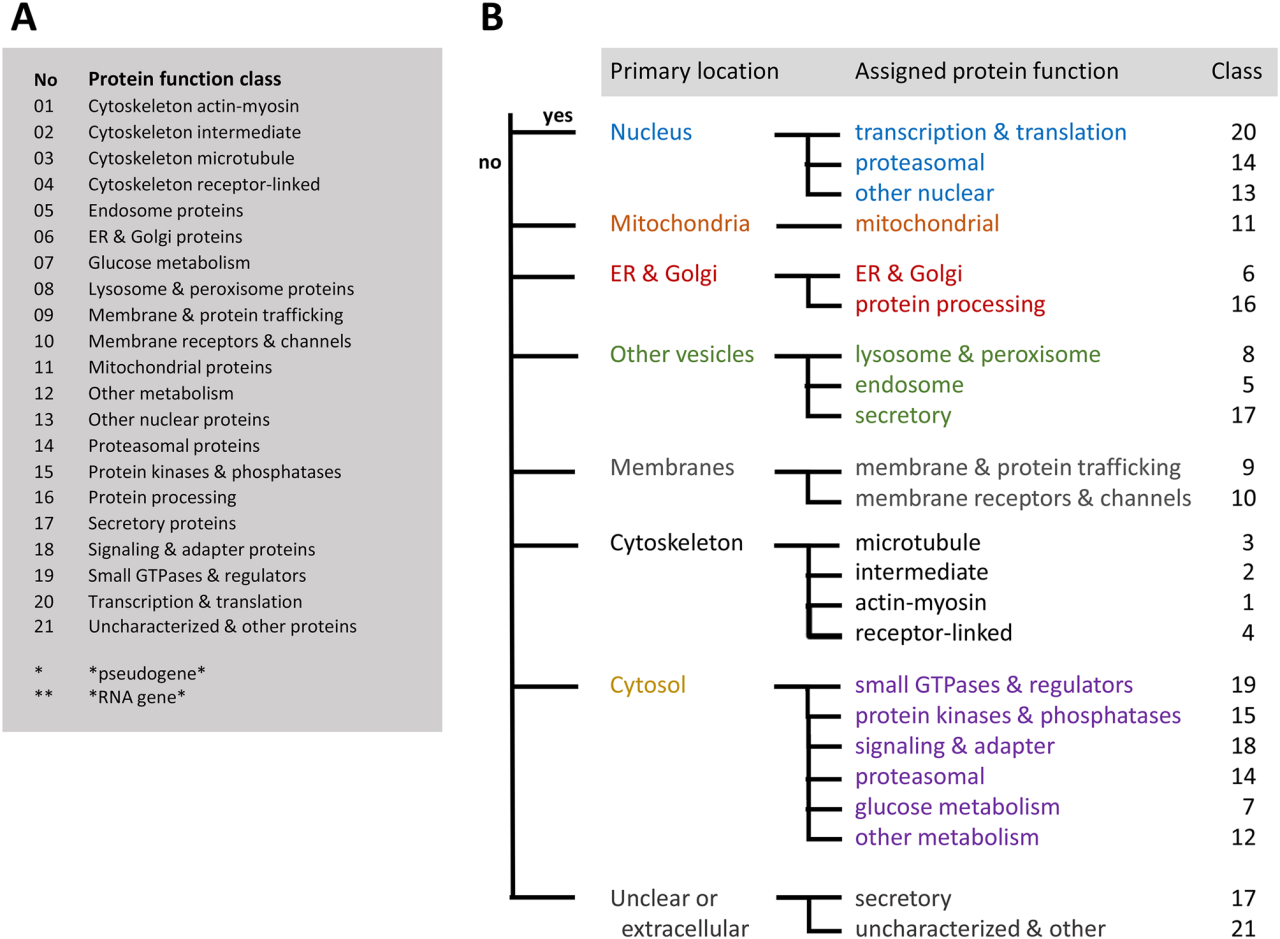
Classification scheme and decision tree for gene and protein assignment to 21 function classes. Assignment was based on primary subcellular localization of the protein and its assumed function according to UniProt-KB. (**A**) Class numbering in alphabetical order. (**B**) Hierarchical decision tree.

Considering that the previously published (phospho)proteomics profiles of highly purified platelets from 22 healthy subjects in 6 cohorts were generated by the same analytical workflow^24–29^, we decided to integrate these datasets (Suppl. Figure 1A). Primary sources of these datasets are listed in Table 1. The resulting, merged human platelet proteome—one of the largest described so far—contained a total of 5,211 identified proteins, of which 80% were present in at least 2 cohorts (Suppl. Datafile 2). For 3,629 of these proteins, also copy numbers per platelet were present. In order to obtain a useful knowledgebase, we then categorized these proteins into 21 classes, based on intracellular localization and function (Fig. 1A). For an objective classification, we used a dichotomous decision scheme together with human UniProt-KB assignments regarding the supposed primary location and/or function of that protein (Fig. 1B). Highest fractions of identified proteins were seen in the following classes (Suppl. Figure 1B): C_20_ (transcription & translation, *n* = 488 proteins), C_12_ (other metabolism, *n* = 475), C_18_ (signaling & adaptor proteins, *n* = 471), C_11_ (mitochondrial proteins, *n* = 455), and C_10_ (membrane receptors & channels, *n* = 327). Distribution profiles of the 3,629 proteins with copy numbers (Suppl. Figure 1C) showed highest abundance and gene expression levels of the classes: C_01_ (cytoskeleton actin- myosin), C_07_ (glucose metabolism) and C_04_ (cytoskeleton receptor-linked). This clustering analysis hence underscored the importance in platelets of signaling, mitochondrial and cytoskeletal proteins^2^.

**Table 1:** Accessibility per proteome cohort of website link (a), used raw datasets (b) and deposited spectral data (c).

*Cohort 1*^24^
a. https://ashpublications.org/blood/article/120/15/e73/30645/The-first-comprehensive-and-quantitative-analysis
b. Supplemental Table S2 and S3: identified phosphopeptides and proteins
c. Pride repository (http://www.ebi.ac.uk/pride/), accessions 22201-22203, 22,206
*Cohort 2*^25^
a. https://ashpublications.org/blood/article/123/5/e1/32883/Time-resolved-characterization-of-cAMP-PKA
b. Supplemental Table S3 and S4: identified phosphopeptides and proteins
c. ProteomeXchange repositories PXD002883 and 10.6019/PXD002883
*Cohort 3*^26^
a. https://ashpublications.org/blood/article/129/2/e1/36101/Temporal-quantitative-phosphoproteomics-of-ADP
b. Supplemental Table 1 and 2: identified phosphopeptides and proteins
c. ProteomeXchange repository PXD001189
*Cohort 4*^27^
a. https://www.ncbi.nlm.nih.gov/pmc/articles/PMC5054341/
b. Supplemental Table 1 and 2: identified phosphopeptides and proteins
c. ProteomeXchange repositories PXD002883 and 10.6019/PXD002883
*Cohort 5*^28^
a. https://www.nature.com/articles/s41598-020-68379-3#Sec25
b. Datafile S1 and Datafile S2: identified phosphopeptides and proteins
c. ProteomeXchange repository PXD016534
*Cohort 6*^29^
a. https://ashpublications.org/blood/article/114/1/e10/26099/Platelet-membrane-proteomics-a-novel-repository
b. Table S4: list of proteins and peptides
c. Pride repository (http://www.ebi.ac.uk/pride/init.do), accessions 8127–8129
*PLT and MGK transcriptomes*
a. https://doi.org/10.3324/haematol.2019.238147
b. Transcript levels: https://blueprint.haem.cam.ac.uk/mRNA/
c. Deposited at BioRxiv https://doi.org/10.1101/764613

### Relevant genome-wide transcriptomes of platelets and megakaryocytes

**Figure 2:**
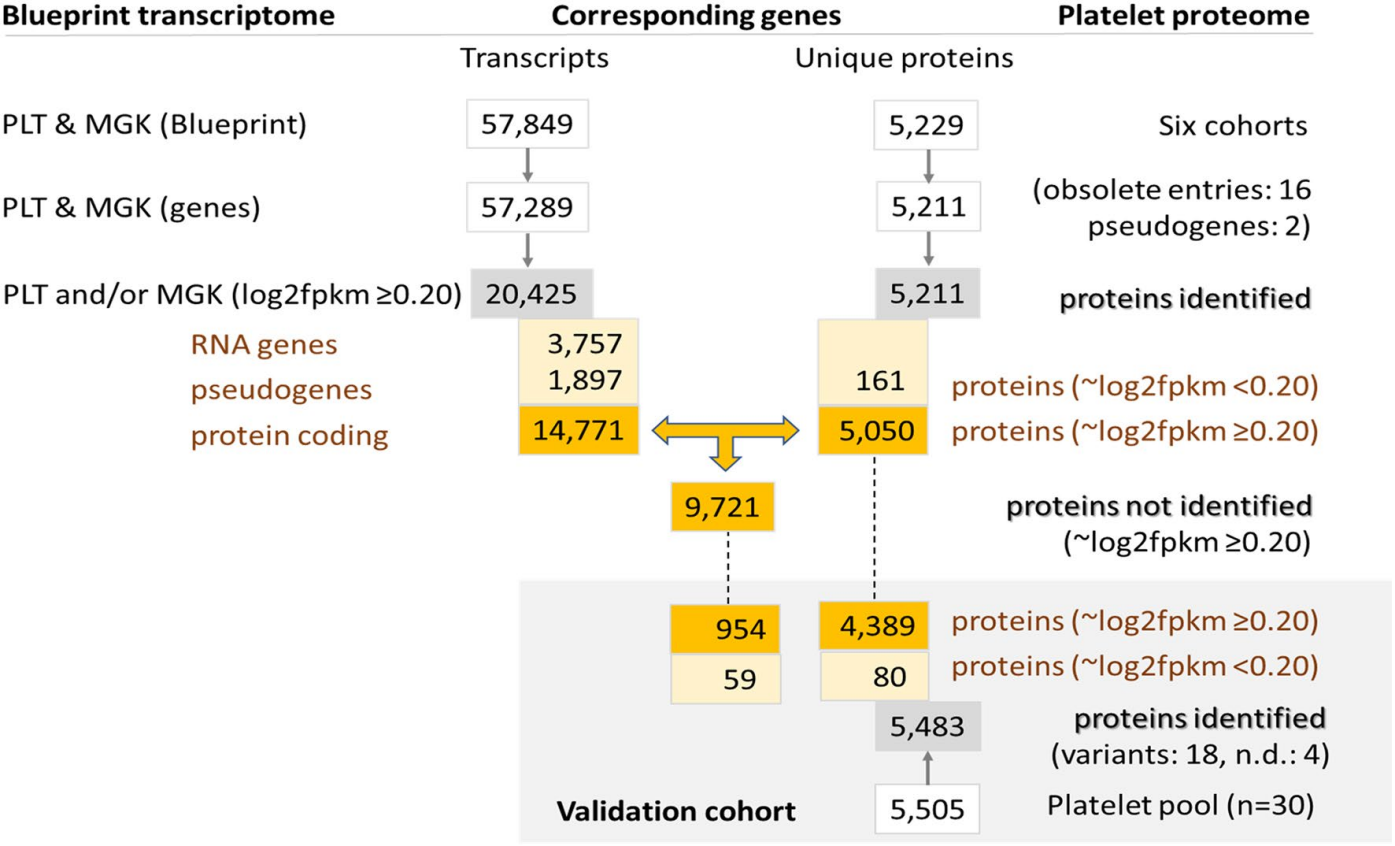
Dataflow of numbers of transcripts of proteome proteins. Relevant transcripts were defined as those of log2fpkm ≥ 0.20. Identified proteins refer to proteins present in the combined proteome from six cohorts. Non-identified proteins refer to proteins with relevant transcript levels in the combined PLT and MGK transcriptome. Data from validation cohort are also indicated.

**Figure 3:**
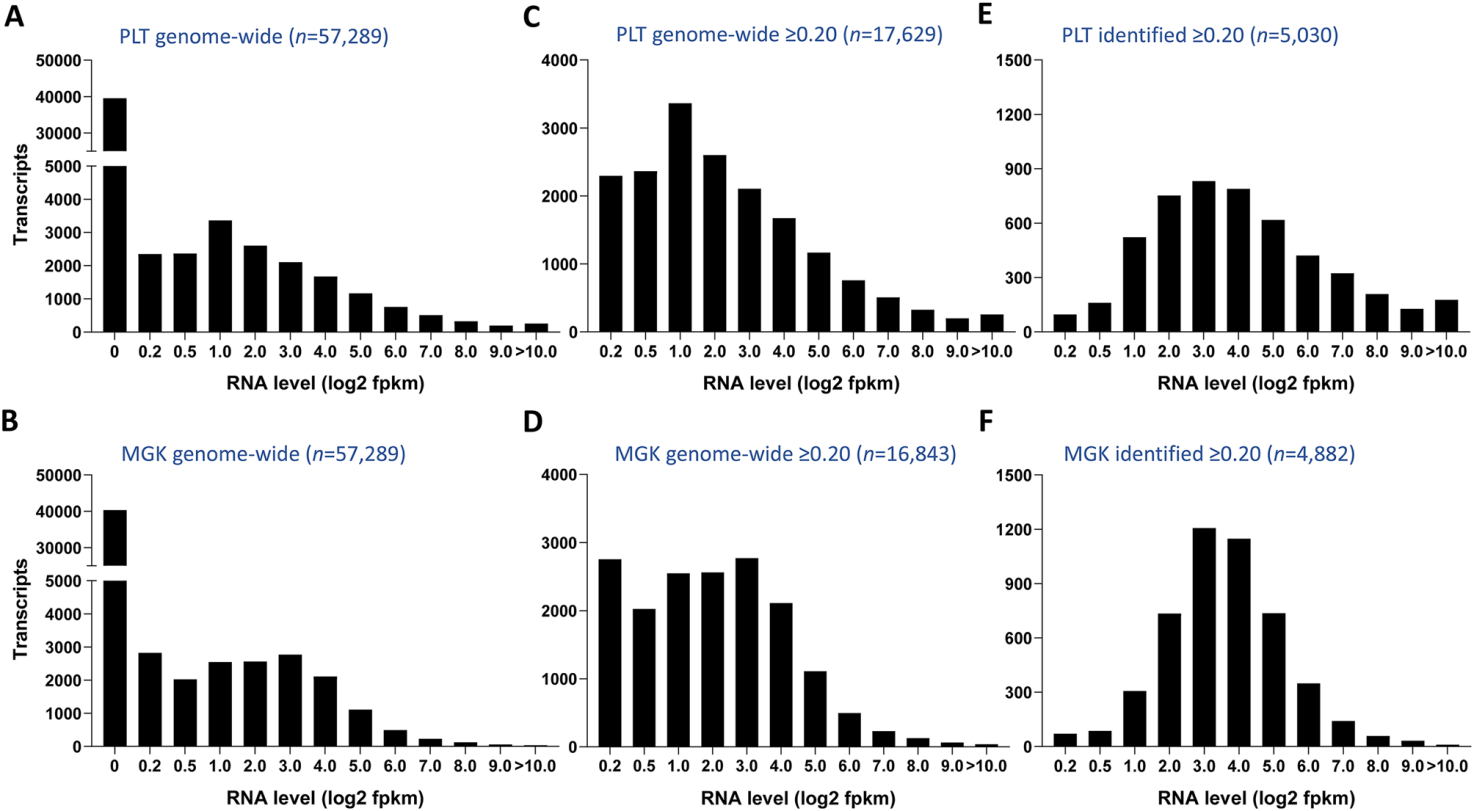
Histograms of RNA levels in transcriptome of platelets (PLT) or megakaryocytes (MGK). (**A**,**B**) Distribution of all 57,289 genome-wide transcripts. (**C**,**D**) Distribution of all relevant transcripts (log2fpkm ≥ 0.20) for PLT (*n* = 17,629) or MGK (*n* = 16,843). (**E**,**F**) Distribution of protein-coding transcripts, as identified in the proteome, for PLT (*n* = 5,030) or MGK (*n* = 4,882). Levels of RNA expression (log2fpkm) were binned as < 0.20, 0.20–0.50, 0.50–1.00, 1.00–2.00, etc. For flow of numbers of transcripts and proteins, see Fig. 2.

Based on well-purified human platelet and megakaryocyte preparations, the Blueprint consortium^30,31^ has recently generated one of the largest databases with genome-wide, quantitative information on a total of 57.8 k transcripts in either cell type (Fig. 2, for source see Table 1). Examination of the distribution pattern of all gene-linked transcripts indicated that 37.2 k of these were essentially absent (log2fpkm 0.02–0.03 ± 0.03, mean ± SD) in platelets (Fig. 3A) and megakaryocytes (Fig. 3B). The residual presence of ~ 20 k expressed transcripts supports earlier analyses of the comparative transcriptomes of blood cells^19^. We then combined these Blueprint datasets with the combined proteome data to come to a draft full platelet proteome.

Based on a low threshold of log2fpkm ≥ 0.20 for relevant expression levels (see below), we obtained a defined set of 20.4 k transcripts, which was taken to assemble the relevant transcriptomes for platelets (17.6 k) and megakaryocytes (16.8 k). Comparison between cell types gave a same distribution pattern (*p* > 0.10, χ^2^) for platelets and megakaryocytes (Fig. 3C,D). Filtering for transcripts of the 5.2 k identified platelet proteins, again resulted in similar distribution patterns (Fig. 3E,F). In either cell type, the lower level transcripts (log2fpkm < 1.00) were under-represented in comparison to the unfiltered genome-wide distribution (*p* = 0.049, χ^2^).

Correlational analysis learned that the platelet and megakaryocyte transcriptomes were highly correlated; this was the case for both the 57.3 k genome-wide transcripts (log2fpkm ≥ 0.00, R = 0.85, β > 0.99) and the 20.4 k transcripts with relevant expression levels in either/both cell types (log2fpkm ≥ 0.20, R = 0.75, β > 0.99; Suppl. Figure 2A, B). This markedly revealed high similarity of the RNA species composition in human platelets and megakaryocytes. Concerning different RNA biotypes, this correlation remained high, when extracting only the protein-coding genes (14.8 k, R = 0.75, β > 0.99), but it reduced for the 3.8 k RNA genes and 1.9 k pseudogenes (R = 0.43–0.54) (Suppl. Figure 2C-E).

For justification of the relevant transcript threshold for protein expression, we reduced this further from log2fpkm 0.20 to 0.15; this resulted in inclusion of no more than 8 extra proteins from the combined proteome, half of it being plasma-derived proteins and the other half with minimal copy numbers. This indicated that log2fpkm of 0.20, although arbitrary, provides a reasonable cutoff value for transcripts resulting in measurable proteins.

Using the combined knowledgebase of platelets and megakaryocytes, we assessed which of the 20.4 k expressed transcripts (log2fpkm ≥ 0.20) were also present in the 5.2 k platelet proteome (Fig. 2). It appeared that the majority of proteins had relevant transcription levels. In 19 of the 21 protein function classes only 1.6% of the protein transcripts were below the cut-off (77/4,907 with log2fpkm 0.04 ± 0.05, mean ± SD, *n* = 19) (Table 2). However, in the classes C_02_ (cytoskeleton intermediate) and C_17_ (secretory proteins), percentages of below cut-off were much higher, amounting to 58% and 24%, respectively.

**Table 2:**
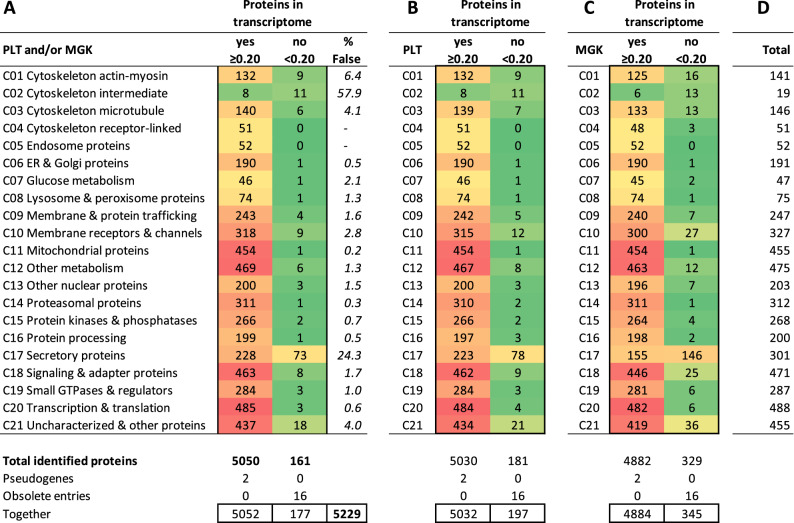
Identified proteins in proteome in comparison to relevant transcriptome of platelets (PLT) and/or megakaryocytes (MGK).

Indicated per function class are numbers of proteins with relevant (log2fpkm ≥ 0.20) or no relevant (log2fpkm < 0.20) mRNA expression. Analyzed were the combined PLT/MGK transcriptome (**A**), as well as the separate PLT (**B**) and MGK (**C**) transcriptomes. For the total of 5,232 identified proteins in the proteome, 2 appeared to be encoded by pseudogenes, and 16 were designated as obsolete entries in UniProt-KB. Also given are percentages of proteins without relevant expression level (% false). (**D**) Total numbers of assigned proteins per class independent of transcript level.

Given the analysis above, we considered that the combined platelet and megakaryocyte transcriptome (either log2fpkm ≥ 0.20) may provide the most extensive list of mRNAs that can be translated into proteins. To evaluate this, we performed the same analysis as above for the platelet-only transcriptome. This resulted in a number of 'false' assignments of 181 (Table 2). For the megakaryocyte-only transcriptome data, this number increased to 329. Accordingly, the combined list of relevant platelet and megakaryocyte transcripts appeared to provide the best overlap with the proteomics dataset. By confining to proteins with relevant mRNA expression, the identified platelet proteome was therefore set at 5,050 proteins.

### Comparison of (non-)identified parts of the platelet proteome

We then reasoned that starting from the genome-wide transcriptome of platelets and megakaryocytes (log2fpkm ≥ 0.20), it was possible to construct a 'full' theoretical platelet proteome and compare this with the identified platelet proteins. By thus comparing the identified proteins with the transcripts of protein-coding genes, we could calculate the remaining, non-identified part of the proteome at 9,721 proteins, *i.e.* 66% of all mRNA transcripts (Suppl. Figure 3A). Based on this analysis, the majority of the 14.8 k proteins in the theoretical proteome was still absent in the current platelet proteomes. A similar number of 14.3 k was obtained when only including the relevant transcripts of platelets (Suppl. Figure 3B,C).

**Figure 4:**
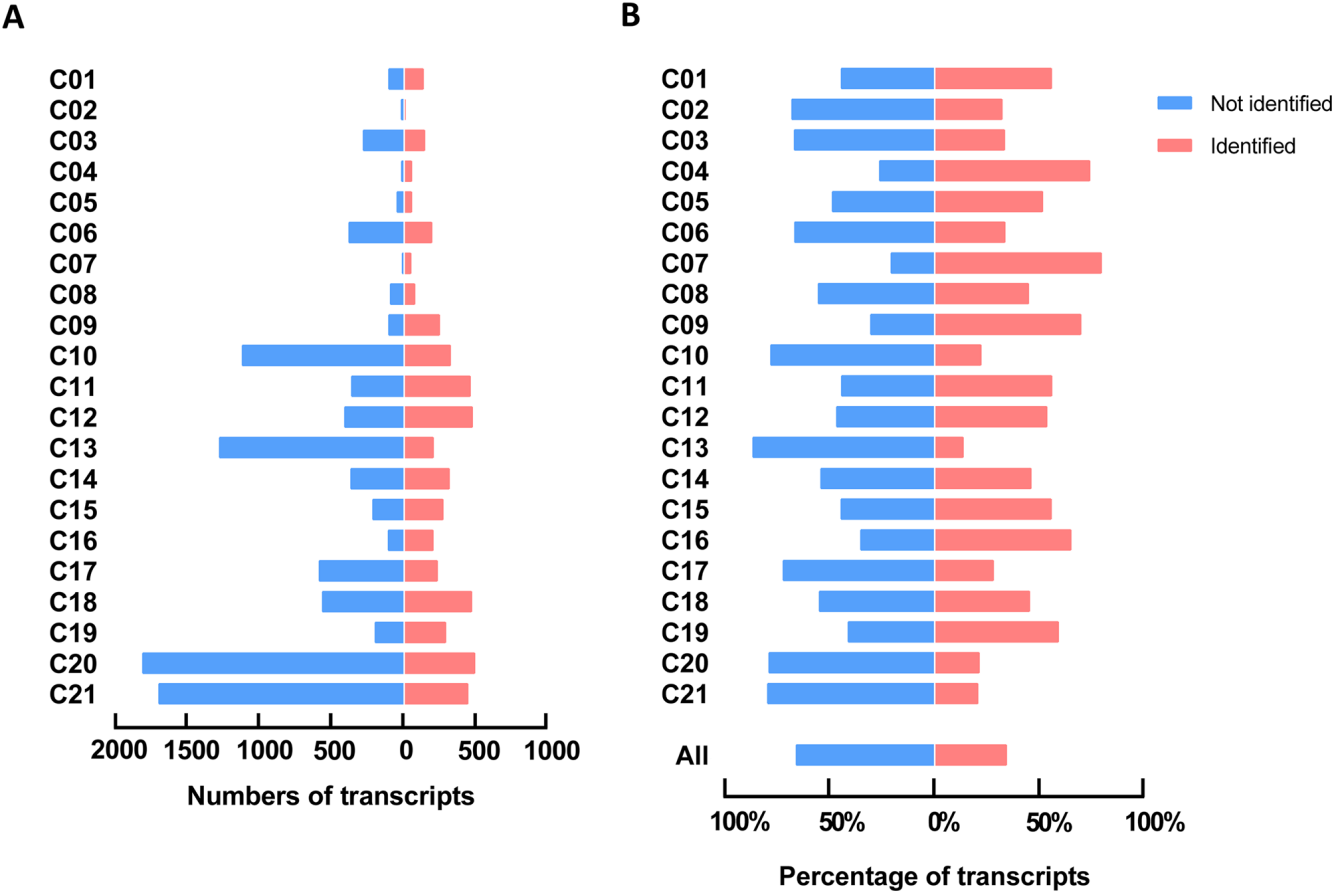
Transcript distribution of identified and not identified proteins in the platelet proteome per function class. Examined were all relevant protein-coding transcripts (log2fpkm ≥ 0.20) of the combined relevant PLT/MGK transcriptome, with separation of identified proteins (*n* = 5,050) and not identified proteins (*n* = 9,721). For full data, see Suppl. Figure 3. (**A**) Numbers of transcripts numbers per function class. (**B**) Percentage distribution of transcripts per function class.

Detailed examination of the genes for which no protein products were detected revealed marked differences between function classes (Fig. 4A,B). Highest numbers and percentages of transcripts of the 'missing' proteins were obtained for: C_20_ (transcription & translation, *n* = 1,795), C_21_ (uncharacterized and other proteins, *n* = 1,683), C_13_ (other nuclear proteins, *n* = 1,269), C_10_ (membrane receptors & channels, *n* = 1,112), C_17_ (secretory proteins, *n* = 583), and C_18_ (signaling & adapter proteins, *n* = 561). This prompted us to investigate the reasons for these inter-class differences in coverage of the identified proteome.

### Restraining factors for a complete platelet proteome

Acknowledging current mass-spectrometry limitations (see Suppl. Methods), we hypothesized that absence of mRNA products can be explained by three restraining factors: (i) low protein copy number, (ii) low mRNA level, and/or (iii) retaining of a protein in the megakaryocyte perinuclear region. The annotated platelet and megakaryocyte transcriptome knowledgebase allowed us to estimate these restraining factors.

**Figure 5:**
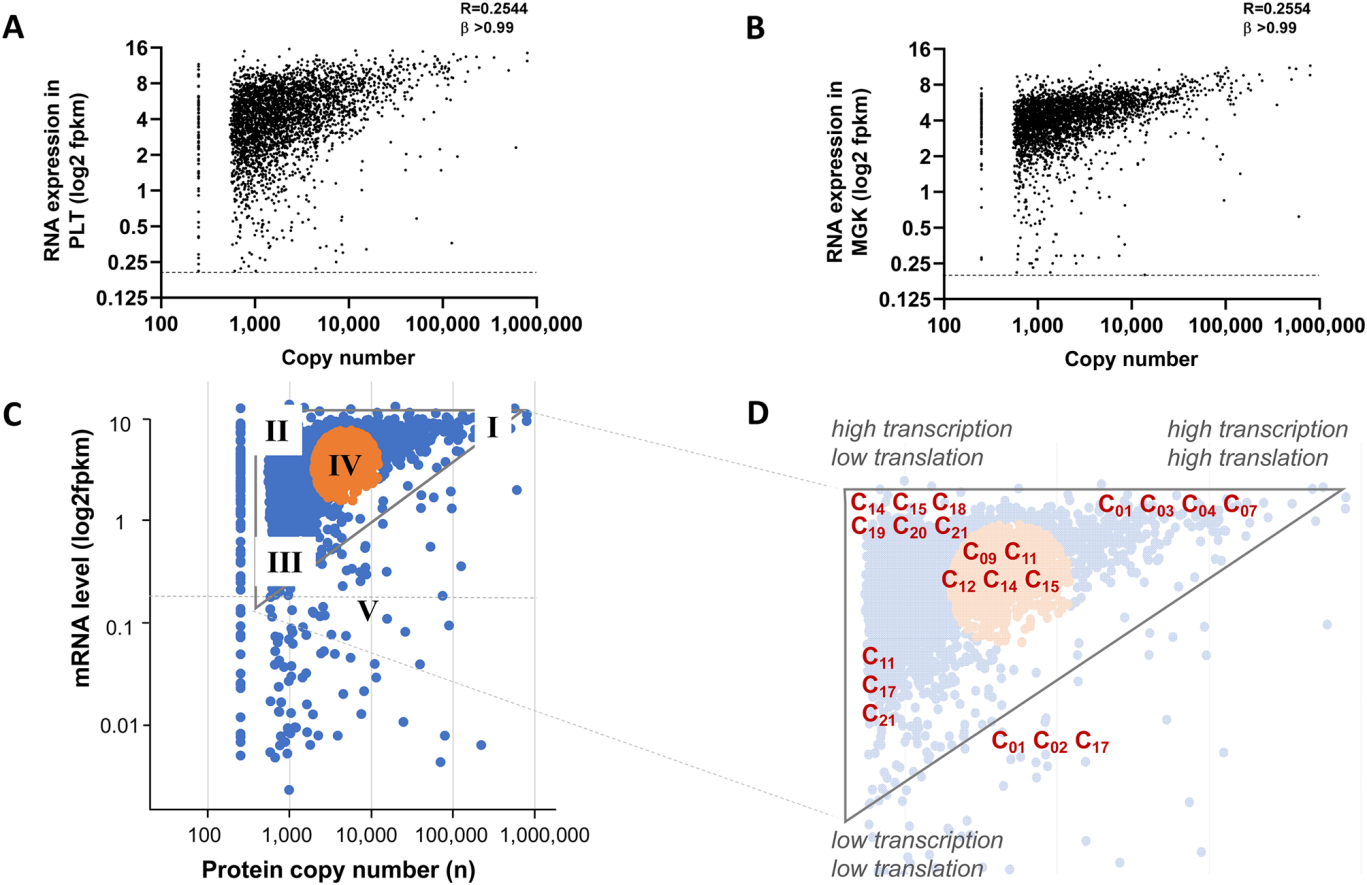
Comparison of protein copy numbers with mRNA levels and class-based analysis. (**A**,**B**) Protein copy numbers compared per gene to transcript levels (log2fpkm) for datasets of platelets (PLT, *n* = 3,519) (**A**) or megakaryocytes (MGK, *n* = 3,442). (**B**) Note triangular space, with low-abundance proteins (< 500 copies/platelet) were normalized to 150 copies. (**C**,**D**) Over-representation of protein function classes in quantitative proteome-transcriptome space per predefined area (I–V). Area I is considered to represent a condition of high translation (high mRNA level) and high transcription (high copy number); area II of high translation and low transcription; area III of low translation and transcription, and area IV an intermediate condition. Area V represents proteins without relevant transcript levels in PLT. Transcriptome-proteome triangle with analyzed areas (**C**). Enlarged space indicating function classes (C_01_-C_21_) with significant over-representation per area. Statistics in Suppl. Table 1.

The relation between platelet copy numbers and transcript levels is still unclear^32,33^. To reassess this issue, we compared the relevant Blueprint transcriptome (log2fpkm ≥ 0.20) with the 3.5 k proteins with known copy numbers. Correlative scatter plots showed a marked triangular pattern (Fig. 5A,B). This pattern indicated that the abundance of a protein was restricted by, but was not otherwise dependent of the transcript level. Given the high similarity of the platelet and megakaryocyte transcriptomes, this implied that the megakaryocytic mRNA levels in fact maximized the extent of protein expression in platelets.

To examine this further, we defined five regions in the proteome- transcriptome space, labeled as areas I-V (Fig. 5C). For each of 3.5 k quantified proteins, we performed a modeling analysis per function class in Matlab. This modelling revealed that—regardless of the use of platelet or megakaryocyte plots—several classes were significantly over-represented (*p* = 10^−2^ to 10^−10^) in some of these areas (Suppl. Table 1). As illustrated in Fig. 5D, for area I (high copy number and high mRNA), four classes were over-represented (i.e., cytoskeletal and glucose- metabolism proteins, *p* < 10^–2^). For the areas II and III with low copy numbers ('low translation'), six and three classes were over-represented, respectively (*e.g*.*,* signaling-related, proteasomal, transcriptional and mitochondrial proteins). Thus, the classes accumulating in areas II-III appeared to be enriched in proteins with low copy numbers, irrespective of their corresponding transcript levels. Area V (low transcript levels) was enriched in keratin-like and secretory proteins (classes C_02_ and C_17_); and area IV of medium mRNA levels contained most of the remaining classes.

**Figure 6:**
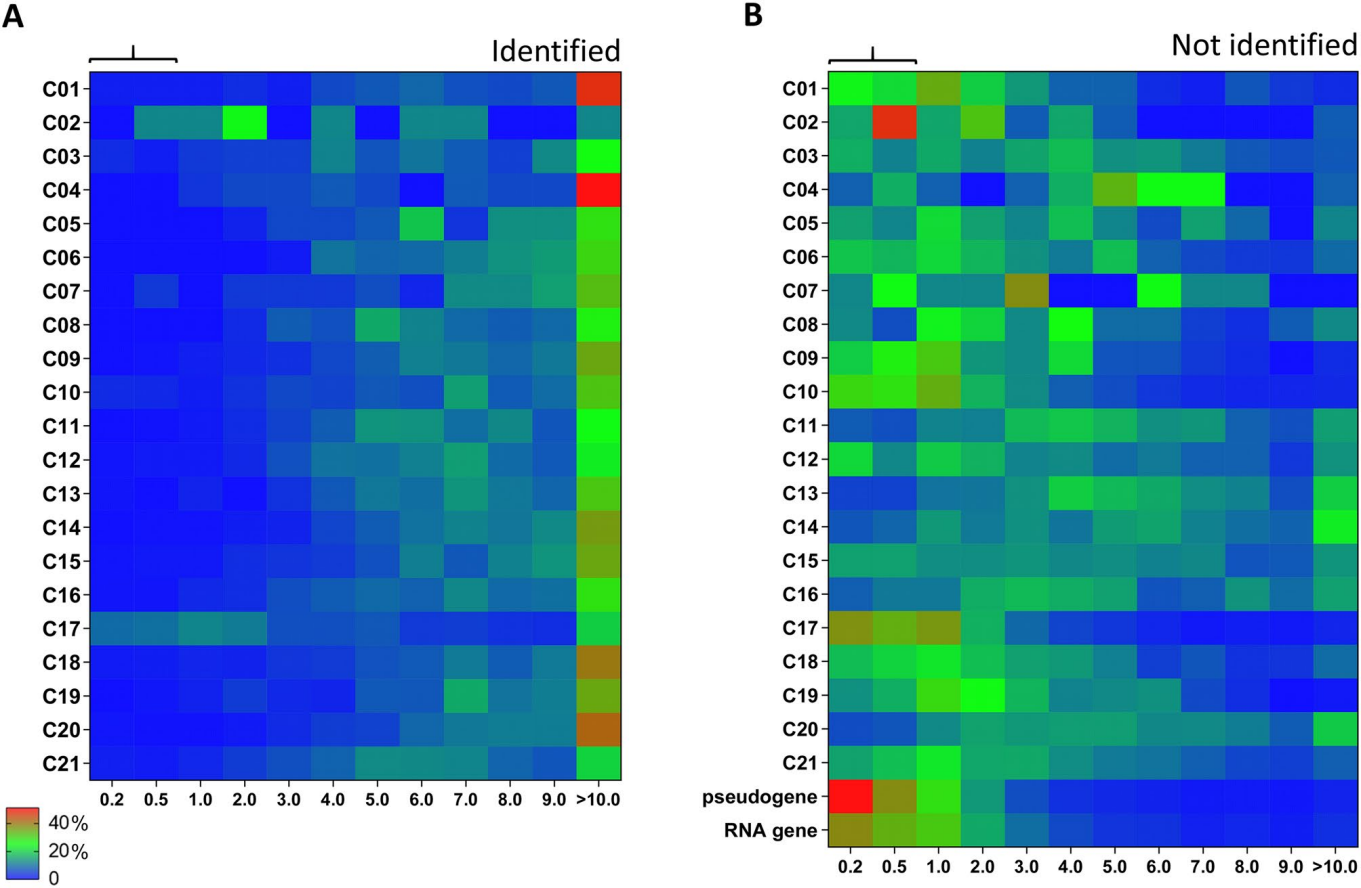
Distribution profile of relevant transcripts of per protein function class. For the relevant platelet transcriptome (*n* = 17,629), heatmaps were constructed of percentual distribution of transcript levels per function class (rainbow colors; blue = low, red = high). (**A**) Heatmap for transcripts of identified proteins (*n* = 5,030). (**B**) Heatmap for transcripts of non-identified proteins (*n* = 9,267); furthermore RNA genes (*n* = 2,480) and pseudogenes (*n* = 852). Expression levels (log2fpkm) were binned as 0.20–0.50, 0.50–1.00, 1.00–2.00, etc. For numbers of transcripts, see Suppl. Figure 3.

To categorize the low-level mRNAs, we examined the transcript level distributions per class, in which we separated the identified and non-identified parts of the theoretical proteome. Overall, the majority of the identified proteins showed relatively high corresponding transcript levels, regardless of their function class (Fig. 6A). On the other hand, the low-level mRNAs (log2fpkm 0.20–1.00) were enriched in the non-identified proteome (median *p* = 0.0005) (Fig. 6B). This held for 12 out of 21 classes, where transcripts of non-identified proteins appeared to be of a lower level.

To examine the low-level transcripts in these 12 classes, we searched for common elements (*n* ≥ 10) in protein names. Examples are: for C_01_: 'actin' or 'myosin'; for C_03_: 'centromere', 'centrosomal' or 'dynein'; for C_06_: 'AP1-3 complex subunit', 'Golgi' or 'trafficking protein particle' (Table 3). Close examination showed that, for all 12 classes with > 20% low-level mRNAs, the same > 20% also applied for elements of the non-identified proteome (Suppl. Table 2). As apparent from the listed most abundant transcripts of elements in almost all classes, the non-identified protein segments contained multiple isoforms or subunits of complexes that were also present in the identified segments, although the former had lower-level mRNAs (Table 3). Furthermore, sets of proteins seemed to be missing in almost all elements.

**Table 3:**
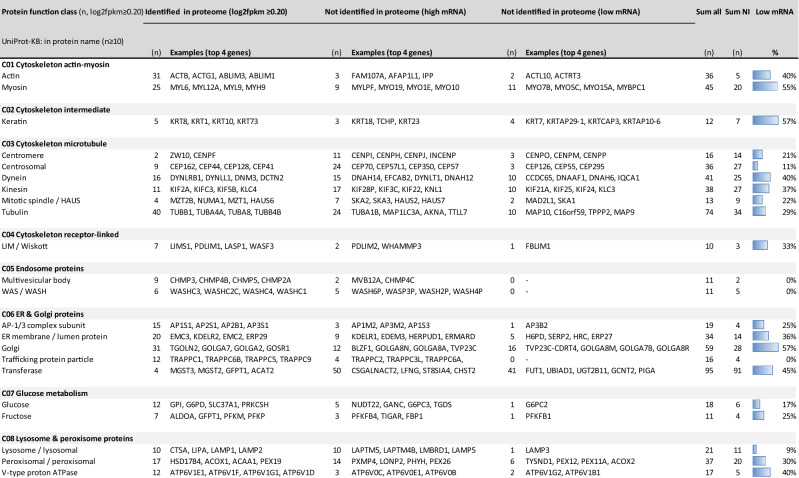

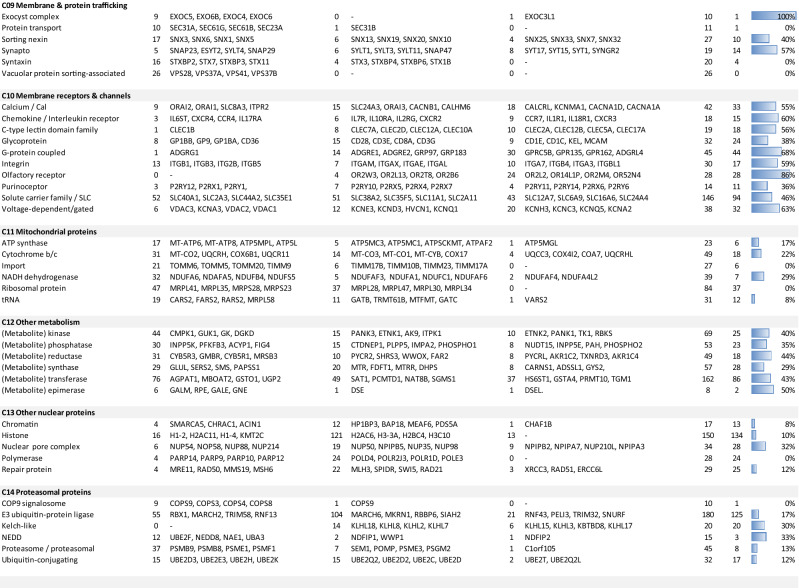

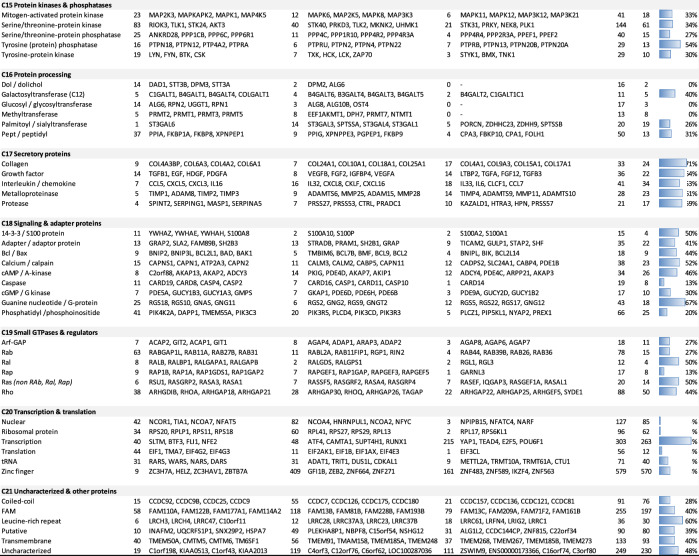
Subgroup analysis of non-identified proteins (n = 9,721) of the relevant PLT/MGK transcriptome.

Per function class (C_01_–C_21_), the transcriptome database was searched for common elements in protein names ('actin', 'myosin'), and frequency was recorded as identified in the proteome, or not identified with a separation into high mRNA (log2fpkm > 1.00) or low mRNA (log2fpkm 0.20–1.00). Top-4 of most abundant transcripts were listed per element. Further indicated per element: numbers of all transcripts (Sum all), and numbers of transcripts not identified in proteome (Sum NI).

As a third restraining factor, we examined protein retainment in the megakaryocyte, by reasoning that in particular (peri)nuclear proteins will not move into a shedding proplatelet. This applied for the classes C_20_ (transcription & translation), C_13_ (other nuclear proteins) and C_03_ (cytoskeleton microtubule), containing multiple centromere/mitotic spindle proteins (Fig. 6A). Hence, these three classes were listed as providing additional explanation for low identification in the proteome (Suppl. Table 2).

### Prediction model of the total platelet proteome

**Figure 7:**
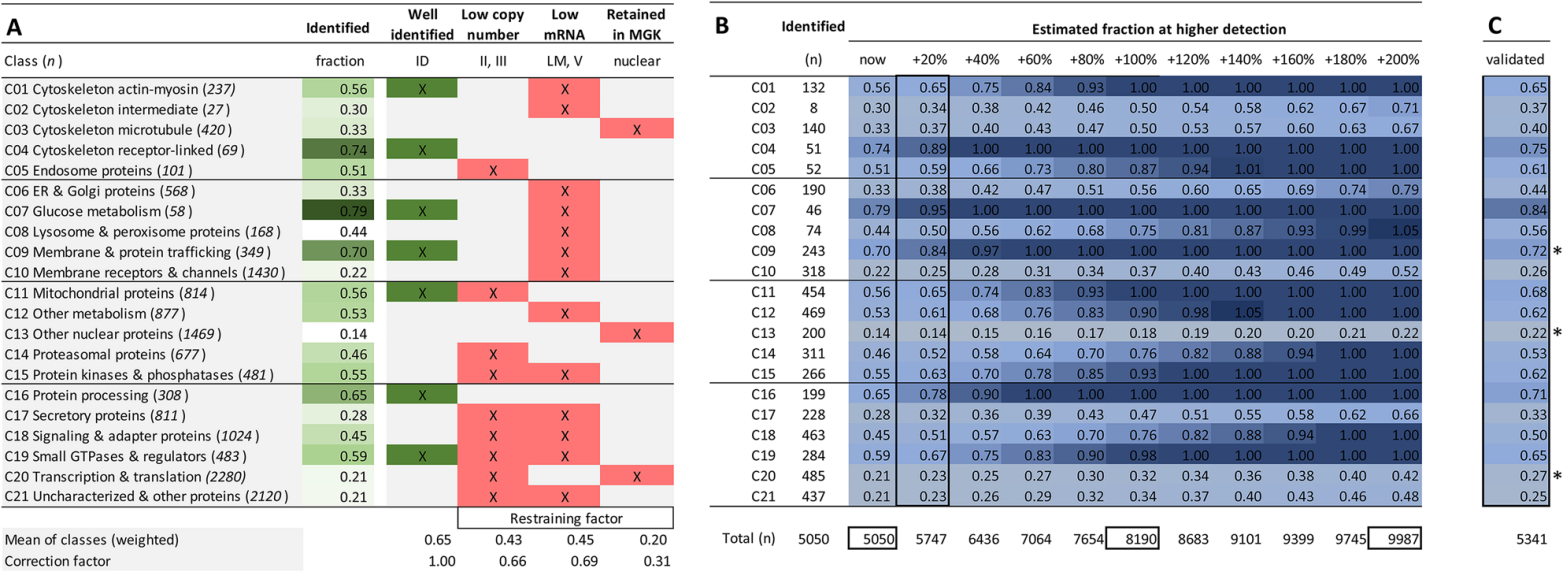
Restraining factors per function class and prediction model of full platelet proteome. Analysis of non-identified proteins (*n* = 9,721) from the relevant, combined PLT/MGK transcriptome per function class. Full dataset is provided in Suppl. Table 2. (**A**) Fraction of identified proteins in green. Well-identified classes with fractions > 0.55 labeled as ID. Indicated in red are each of three restraining factors per class: (i) over- represented low copy number (areas II-III in Fig. 5D), (ii) low mRNA level (area V, LM = low mRNA > 45%); (iii) retainment in megakaryocyte (peri)nucleus upon platelet shedding. Bottom: means of identified fractions (weighted for the presence of multiple factors); and correction factor in comparison to 'well-identified'. (**B**) Based on identified proteins (*n* = 5,050), modelled prediction of increased identification of missing proteins per class at higher proteomic detection. Shown per class are fractions of total relevant transcripts (heatmapped), and total expected proteins (bottom line). (**C**) Validation of prediction model based on novel proteome with 5,341 identified proteins.

We then established an matrix for determining the three restraining factors per class (Fig. 7A). This matrix was then used to calculate weighted mean values of the fractions of identified proteins grouped per factor. The fractions of identified proteins for (i) low copy number, (ii) low mRNA > 20%, and (iii) retainment in megakaryocytes, amounted to 43%, 45% and 20%, respectively. For all other classes, the average fraction of identified proteins was 65% (Fig. 7A). By ratioing, this resulted in correction factors (0.66, 0.69 and 0.31, respectively) for class predictions of the likeliness that additional proteins would appear in an enlarged proteome (Fig. 7B).

Summarizing, the prediction model indicated a greatly enlarged size of the platelet proteome up to 10 k proteins at a 1- or twofold higher detection efficacy. Markedly, apart from a consistent underrepresentation of classes of (peri)nuclear proteins (C_03_, C_13_, C_20_), the model also predicted that a poor detection of proteins in the classes: C_10_ (membrane receptors & channels), C_17_ (secretory proteins), and C_21_ (uncharacterized & other proteins).

### Proteome model validation

For validation of the model, we performed a new proteomic analysis with pooled platelets from 30 healthy subjects and the newest mass spectrometers. The obtained proteome included 4,389 of the previously identified proteins with relevant transcripts, as well as 954 previously not identified proteins (Fig. 2; details in Suppl. Datafile 3). Of additional 139 proteins without relevant transcript levels (log2fpkm < 0.20), the majority of 70% again appeared in C_02_ (intermediate cytoskeleton, *n* = 15, 11%) and C_17_ (secretory proteins, *n* = 81, 58%). This underscored the earlier observation that keratins and plasma proteins are present in the proteome of platelet samples.

Concerning the 954 novel obtained proteins, only small fraction of 3.8% showed low transcript levels with log2fpkm 0.20–1.00. Heatmap representation showed an similar distribution profile for all classes (Suppl. Figure 4). Markedly, inclusion of the novel proteins agreed with the prediction model for the majority of classes (Fig. 7C). Interestingly, higher than expected were the novel proteins for C_20_ (transcription & translation, additional 139 proteins) and C_13_ (other nuclear proteins *n* = + 121); lower were those of C_09_ (membrane proteins, *n* = + 7).

### Coverage of genes associated with hemostasis and thrombosis

**Figure 8:**
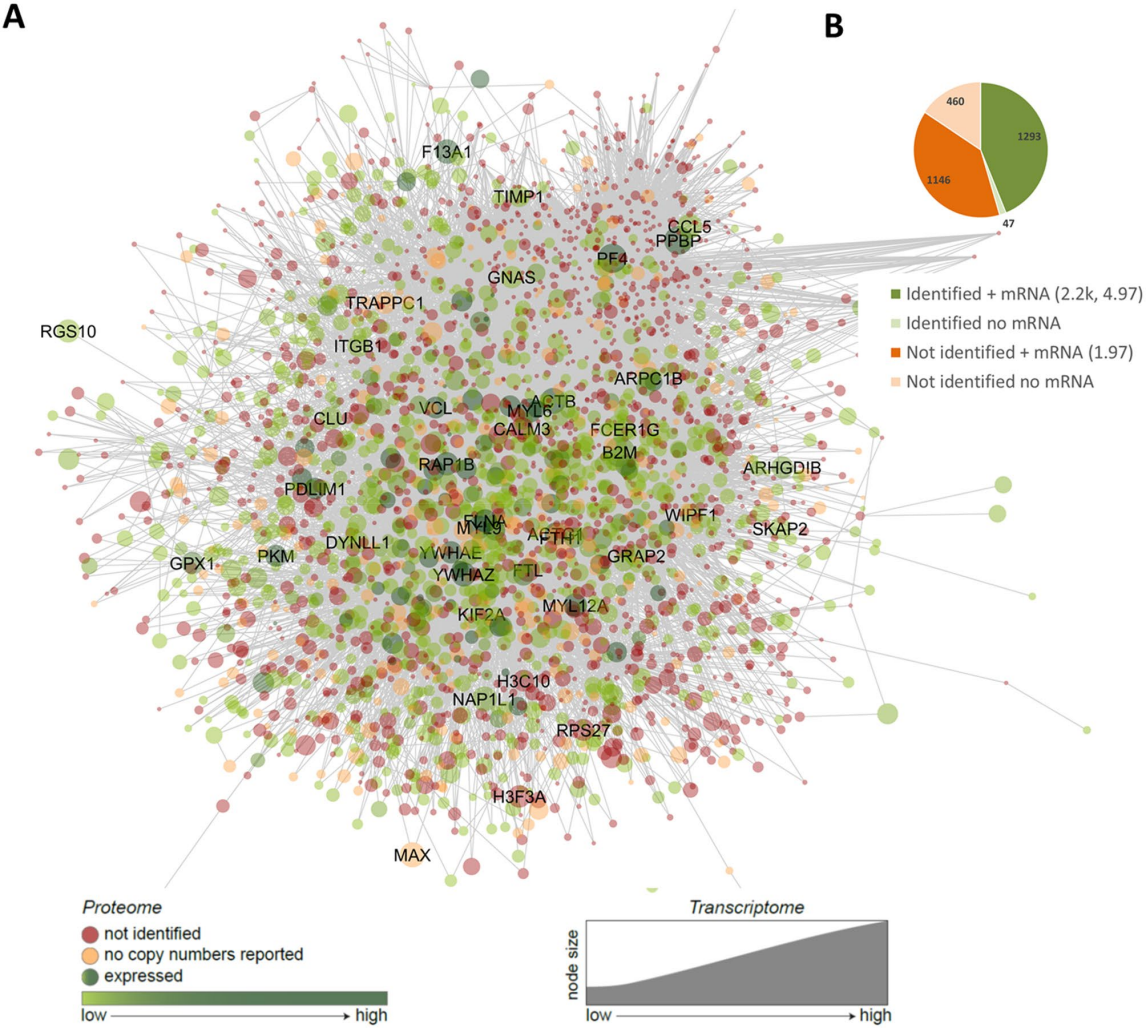
Network-based potential roles of (non)identified proteins in platelet proteome in arterial thrombosis and hemostasis. Using a published meta-analysis of mouse genes in thrombosis and bleeding, the network was built in Cytoscape, containing 267 core genes (bait nodes), 2679 new nodes, connected by 19.7 k interactions^15^. (**A**) Redrawn network visualization with color-coded proteins identified (green) or not identified (red) in the platelet proteome, with relevant transcript levels (node size, log2fpkm). Names are listed of 40 proteins with highest mRNA expression levels. (**B**) Distribution profile of (non)identified proteins with transcript levels (median copy numbers, median log2pkm). No mRNA = below relevant threshold. Attribute lists are given in Suppl. Datafile 4.

To further establish the clinical relevance of these datasets, we incorporated the identified proteome set into a Reactome-based protein–protein interaction network (267 core proteins and 2,679 new nodes) that was constructed to identify the roles of platelet and coagulation proteins in thrombosis and hemostasis^15^. As shown in Fig. 8, this network incorporated 1.3 k of the identified proteins (median protein copies 2,200, median transcript level log2fpkm 4.97), as well as a set of 1.1 k proteins/transcripts (median log2fpkm 1.97) not present in the combined proteome (Fig. 8A,B). Importantly, of the latter set, 172 proteins were obtained in the proteome of the validation cohort.

To further establish the coverage for platelet-related disorders, we extracted the databases Online Mendelian Inheritance in Man (OMIM)^34^ and Bloodomics^23^ in combination with a recent overview paper^35^ for genes associated with bleeding, thrombocythemia or thrombophilia. This resulted in 138 genes, of which 9 were absent in the platelet transcriptome but present in the proteome (coagulation factor and other plasma proteins), and 5 were absent in both (Table 4). For the remaining set of 124 genes, transcript levels (log2fpkm 4.58 ± 3.70, mean ± SD) and copy numbers (22.8 ± 73.0 k) in platelets were relatively high. Markedly, the majority of these 124 genes encoded for proteins in the classes C_10_ (membrane receptors and channels, *n* = 22), C_17_ (secretory proteins, *n* = 19), C_20_ (transcription & translation, *n* = 12), C_18_ (signaling & adapter proteins, *n* = 10), with a lower presence in the other classes. In accordance with the network analysis, it is likely that many still unknown gene products link to a platelet quantitative or qualitative traits, and hence to bleeding or thrombosis. The near complete coverage of the theoretical platelet proteome for known hemostatic pathways was also checked in the Reactome database (not shown).

**Table 4:**
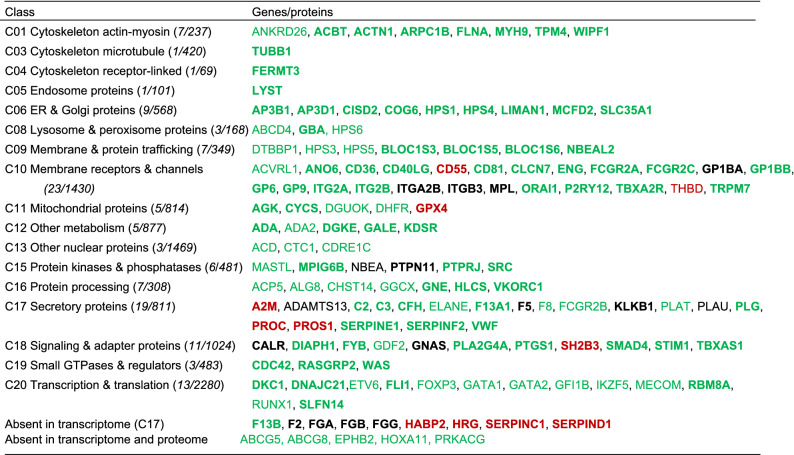
Platelet-expressed proteins in whole-genome transcriptome implicated in hemostasis and thrombosis.

Listed are per platelet function class genes expressed in the (non)identified platelet proteome, which according to recent OMIM, Bloodomics and overviews^23,34,35^ in man contribute to bleeding, thrombocytopenia or thrombophilia. Coding as follows. **Bold**: identified in platelet proteome; green: bleeding or thrombocytopenia; red: thrombophilia; black: either reported.

## Discussion

In this paper, we integrated in a functional way the human platelet proteome, using data from six cohorts established in the same institute, with the recently composed genome-wide, > 57 k platelet and megakaryocyte transcriptomes from the Blueprint consortium^30^. By UniProt-aided categorization of all relevant transcripts (set at log2fpkm ≥ 0.20) into 21 protein function classes, we were able to generate a first full proteomic map of the sub-cellular, metabolic and signaling molecules in an average human platelet. Importantly, this analysis also provide a reference list of 37.2 k transcripts according to our lists are not or hardly expressed in platelets.

Overall, the manuscript covers six major novel aspects: (i) for the first time we established the full or theoretical platelet proteome based on a state-of-the-art genome-wide platelet and megakaryocyte transcriptome; (ii) using > 57 k transcripts we identified an unexpected high similarity of the quantitative platelet and megakaryocyte transcriptomes (including RNA gene transcripts), in spite of a weak correlation between the protein and transcript levels, providing insight into the distribution of RNA species upon platelet shedding; (iii) based on the systematic protein classification, the collected data provide molecular understanding of the complexity of platelet structures and functions; (iv) based on the established theoretical proteome, we developed and also validated a prediction model for identifying missing proteins in the current proteome sample sets; (v) the combined datasets offer better understanding of protein adhesion and uptake of plasma proteins by platelets; (vi) the combination of quantitative transcriptomes and (partly) quantitative proteomes completes our knowledge of the roles of > 100 genes and proteins in diseases not limited to thrombosis and hemostasis.

Correlational analysis of the 20 k expressed transcripts in platelets and/or megakaryocytes indicated an overall high similarity between the transcriptomes of the two cell types. This particularly held for the 14.8 k transcripts of protein-coding genes (R = 0.75), while the correlation was lower for the 3.8 k RNA genes and 1.9 k pseudogenes (R = 0.43–0.54). Although inter-individual differences are expected, our findings indicate that the majority of mRNA species evenly spread from megakaryocytes to the formed proplatelets, with limited degradation during platelet ageing. The aberrant transcript profiles of pseudogenes and RNA genes, which in general were more abundant in megakaryocytes, may be due to retention or to enhanced degradation of such shorter RNA forms^36^. In agreement with our findings, also other authors presenting smaller-size and not genome-wide datasets (3.5 k proteins and 5.5 k mRNAs), have reported a low correlation between platelet protein and transcript levels^37,38^. This lack of correlation however does exclude a role of altered mRNA and protein levels in platelet-related diseases^21^.

Based on the composition of the genome-wide transcriptomes of platelets and megakaryocytes, we calculated that the current proteome of 5,050 expressed proteins misses approximately 66% of the expected translation products. Highest percentages of missing proteins were seen in the classes C_20_ (transcription & translation 79%), C_21_ (uncharacterized proteins 79%), C_13_ (other nuclear proteins 86%), C_10_ (membrane receptors & channels 78%), C_17_ (secretory proteins 72%), and C_18_ (signaling & adapter proteins 55%). Especially low-level mRNAs (log2fpkm 0.20–1.00) appeared to be missing in the identified proteome, likely giving rise to only low copy numbers of proteins.

Proteomic technologies have been well developed, since the publication of the first draft human proteome, which revealed 17.3 k gene products and 4.1 k protein N-termini^39^. Accordingly, the present set of 5.0 k identified platelet proteins is higher than earlier published proteomes, *e.g.* of mouse platelets of 4.4 k proteins with copy numbers^40^, or of the semi-quantitative 3.5–4.8 k proteins in human platelets^38,41^. Smaller size published platelet sub-proteomes are a 0.1 k secretome^42^, and a 1.0 k sheddome^43^. Regarding platelet transcriptomes, which are more uniformly to construct, other authors have published a similar 20 k size with 16 k transcripts at > 0.3 fpkm^44^.

As a check of the present concept—starting from genome-wide platelet and megakaryocyte transcriptomes to determine the theoretical proteome—we evaluated the proteomes reported in three papers, using the current GeneCards gene designations. The proteomes of platelets from Dengue patients^45^ or from platelet concentrates^46^ were found to contain 93.1% (1,769/1,901) and 98.4% (2,466/2,505) proteins that were present in our protein database. Proteins without relevant transcripts were quite low, 2.1% and 0.1%, respectively. A paper analyzing the proteomes from cord blood and adult peripheral blood platelets^47^ showed lower overlap of 79.9% (3,950/4,941) with the current proteome, supplemented with 16.4% proteins with relevant transcripts and 3.7% (183/4,941) without relevant transcripts in dataset. For the last fraction, it is unclear if residual presence of neonatal transcripts contributes to this higher percentage.

In platelet proteomics, the detection of proteins from blood plasma or other blood cells is a continuous point of attention. Our analysis based on highly purified, washed platelet preparations indicated the invariable present presence of plasma proteins. This can be explained by the fact that platelets exhibit an extensive open canicular system (estimated at 1 vol%) in open contact with the plasma, and furthermore also endocytose plasma proteins. The list includes 73 proteins classified as C_17_ (secretory proteins) without corresponding mRNAs, of which at least fibrinogen and β2-glycoprotein 1 are known to be taken up by platelets^48^. Of note, fibrinogen levels are greatly reduced in the proteome of patients with Glanzmann's thrombasthenia, lacking integrin αIIbβ3. At the other hand, we find that multiple 'plasma proteins' can also be expressed by platelets themselves. Hence, even with the development of quality checks of 'plasma contamination', it may be difficult to rate many secretory proteins as platelet or non-platelet.

Apart from the inevitable presence of plasma proteins in platelet preparations, also other conditions may influence the obtained platelet protein composition. One relevant condition is that of macro-thrombocytopenia (e.g., Bernard-Soulier syndrome), often resulting in more fragile platelets, where obtaining of the high quality platelet preparation is a challenge. Another factor is emperipolesis, such as engulfment of hematopoietic cells by megakaryocytes in malign disorders, also affecting the platelet proteome.

To explain the missing of proteins in the identified proteome, we considered three restraining factors: (i) low protein copy number, (ii) low mRNA level, and (iii) protein retainment in the megakaryocyte perinuclear region. By estimating these restraining factors per protein function class, we calculated the technically achievable proteome of ~ 10 k proteins. The assumption is that improved technical developments will generate larger size proteomes (Suppl. Methods).

For validation of the function class-based prediction model of the remaining part of the proteome, we generated an additional proteomic set, which revealed 1.0 k new proteins in the predicted classes, of which 97% with relevant transcript levels. Interestingly, nuclear-related proteins were more frequently present than was predicted, thus pointing to a more prominent incorporation of (peri)nuclear proteins in megakaryocyte-shed platelets than was anticipated.

The function class-based analysis of (non)identified platelet proteome, based on relevant transcript levels (log2fpkm ≥ 0.20) as well as the listing of 37.2 k genome-wide not expressed transcripts provides novel and detailed information on the presence of protein isoforms, subunits of complexes and metabolic, protein processing and signaling pathways (see Table 3). For instance, regarding the apoptosis-related Bcl/Bax proteins (C_18_) involved in platelet clearance^49^, the isoforms BNIP2, BCL2L1 (BCL-XL or BIM), BAD and BAK1 are present in the current proteome, while also the transcripts of BLC7B, BCL9 and BCL2 are highly expressed. As another example, regarding the glycosyl transferases (C_16_) and epimerases (C_12_) implicated in the surface glycosylation pattern and thereby in platelet survival time^50^, prominently present in the proteome (transcriptome) are GALM, GALE, GNE, C1GALT1 and B4GALT1/3/4/5/6, while C1GALT1C1 (COSMC) is only lowly transcribed.

In this Covid-19 era, our list also provides information on ACE2, BSG and TMPRSS2. In platelets and megakaryocytes, ACE2 expression levels appear to be very low (log2fpkm 0.00–0.03), similar to the levels in other blood cells (https://blueprint.haem.cam.ac.uk/bloodatlas). On the other hand, BSG (basigin) with high transcript levels is present in the platelet proteome, but not the marginally expressed TMPRSS2.

Both network analysis and OMIM-based evaluation of the genes/proteins known to contribute to platelet count, hemostasis and thrombosis showed high coverage by the current platelet proteome and transcriptome dataset. Since still little is known of many of the proteins, the list of 20 k transcripts reveals a wealth of novel information on proteins that will influence platelet structure and function. Knowledge for understanding disease processes is still limited, as prior work from our and other labs describe only small-size alteration in platelet (phospho)proteomes of patients with Scott (*ANO6*)^27^ or Glanzmann (*ITGA2B*)^48^ disorders or with pseudohypoparathyroidism (*GNAS*)^28^. Altogether, this underscores that our approach to define a complete platelet proteome provides a valuable scaffold for further exploring and understanding platelet traits in and beyond thrombosis and hemostasis.

The current approach to define a classified full or theoretical platelet proteome from transcriptomes of platelets and megakaryocytes offers new insights into platelet composition and function, but also has limitations. As discussed above, platelets and megakaryocytes can bind and incorporate proteins from plasma, extracellular matrix or other cells, where the corresponding transcripts can be missing. In case of low transcript levels, copy numbers of proteins in platelets can be too low to be detected by mass spectrometric techniques (for detailed discussion on technical limitations, see supplementary methods). Furthermore, the source (individual healthy, diseased subject) and purification method of platelets and megakaryocytes can influence the specific composition of proteome and transcriptome, especially regarding the more rare molecules. It is noted here, that a subset of proteins expressed at very low copy numbers may be relevant for platelet ontogenesis, but have limited impact on platelet functions.

Earlier analyses indicated that the platelet proteome from healthy subjects is quite stable with < 15% of changes^51^. Similarly, the global platelet proteomes from the few patients, extensively studied so far—such as Albright hereditary osteodystrophy, Glanzmann or Scott syndrome patients—showed only minor changes compared to that of control subjects^27,28,48^. the technical abilities to study this in the future is made in the revised discussion (page 16). In the near future, with the use of roboting techniques allowing higher throughput analysis of large sample sets and with the application of stable isotope markers^17^, we expect to know more on the variable part of the platelet proteome in health and disease.

## Methods

### Subject cohorts and platelet samples

Washed, purified blood platelets were obtained in the same laboratories from six cohorts of healthy control donors, anonymized for medical-ethical reasons after informed consent. For each cohort, platelet samples were freshly isolated from anticoagulated blood by first collecting platelet-rich plasma, and removing plasma by a double wash step. Contamination was < 0.02% for red blood cells and leukocytes, presence of plasma about 1 vol%. Raw proteomic data per cohort are provided in the following papers. Cohort 1 (*n* = 3) in Burkhart et al*.*^24^, cohort 2 (*n* = 3) in Beck et al*.*^25^, cohort 3 (*n* = 3) in Beck et al*.*^26^, cohort 4 (*n* = 2) in Solari et al*.*^27^, cohort 5 (*n* = 8) in Swieringa et al*.*^28^, and cohort 6 (*n* = 3) in Lewandrowski et al*.*^29^. Platelets were always derived from anonymous healthy donors, due to ethics restrictions also not revealing age or sex. New experimental work was approved by the Ethics Committee of Maastricht University and Maastricht University Medical Centre^28^.

The genome-wide Blueprint gene expression data were generated from platelets obtained from venous blood (*n* ≥ 3 per transcript, NHS Blood and Transplant healthy blood donors), and depleted from leukocytes^23,31^. Primary data are public accessible via https://blueprint.haem.cam.ac.uk/mRNA/ or htt ps://blueprint/haem.cam.ac.uk/bloodatlas/. ^31^. Purity of platelets was checked by Sysmex, hemocytometer and from transcriptional signatures. Culturing of megakaryocytes (*n* ≥ 3 per transcript) from cord blood, and check by flow cytometry (CD41 and CD42 double-positive) were as described^19^. Blood samples from healthy volunteers were obtained after full informed consent according to the Declaration of Helsinki.

### Proteomes

In all reported studies, platelet lysates were analyzed according to a common bottom-up mass-spectrometry proteomics approach in the same laboratory. Experiments details are in the original papers^24–29^. Briefly, purified lysed platelets were subjected to a filter-aided sample preparation or ice-cold ethanol precipitation procedure. Isolated proteins were then trypsin-digested in guanidinium HCl or urea and (triethyl) ammonium bicarbonate (incubated over night at 37 °C). For global proteome analysis, complex peptide mixtures were fractionated by high-pH reversed phase chromatography (pH 6 or 8). For detection and quantification of platelet phospho-peptides, an enrichment procedure was included using TiO_2_ beads, followed by hydrophilic interaction liquid chromatography (HILIC) fractionation. Fractions of peptides or phosphopeptides were analyzed by nano-liquid chromatography (LC)-MS/MS using QExactive (QStar Elite) and Orbitrap Velos mass spectrometers. Raw data were processed with Proteome Discoverer, SearchGui and Peptide Shaker implemented with Mascot and Sequest and X!Tandem search algorithms. Spectra were searched against a human UniProt-KB database. For database versions, see the original papers^24–29^. In all cases, a false discovery rate (FDR) of 1% was set.

### Primary data deposits and links

Primary datasets were downloaded per proteome cohort via the website links of Table 1, also providing information on the deposited spectral datasets. In cohort one (*n* = 3 subjects), relative protein abundance levels^52^ were determined in combination with a protein abundance estimate to give protein copy numbers per platelet^51^. In brief, protein copy numbers were assessed based on a normalized spectral abundance factor (NSAF) method. First, absolute quantification information was obtained from a set of 24 reference proteins (providing reference copy numbers), which then was used to correct NSAF indexes and was extrapolated to copy numbers of remaining proteins with known NSAF values.

In cohorts 2–5 (*n* = 3, 3, 2, 8 subjects, respectively), additional proteins were obtained without copy numbers, obtained from either global proteome analysis and/or phosphoproteome analysis^25–28^. In cohort 6 (*n* = 3 subjects), platelet membrane proteins were identified^29^. Presence of individual proteins per cohort is indicated in Suppl. Datafile 2.

### Proteome tabling construction

The summative identified proteins with or without copy numbers, derived from global proteome or sub-proteome/enrichment (phospho-proteins or membrane proteins) analysis, were all checked in UniProt-KD (consulted January 2019—January 2020) and listed per corresponding gene (GeneCards). If no match between UniProt-KD assignment and gene name was found, additional gene databases were consulted (Biomart, Ensembl).

### Transcriptomes

Genome-wide quantitative data of 57,849 transcripts assessed in human platelets and human megakaryocytes were established via a guided procedure by the Blueprint consortium^23,31^. For link to sources, see Table 1. For establishing relevant transcription levels, we used an arbitrary, low expression cut-off of log2fpkm ≥ 0.20, which included lowly abundant transcripts, to include all theoretical proteins presumably with very low levels (Suppl. Datafile 1).

### Functional classification of protein-coding and other transcripts

The knowledge bases GeneCards (consulted January 2019—January 2020) was used to primarily separate protein-coding genes, RNA genes and pseudogenes. GeneCards provides comprehensive information on the annotated and predicted human genes, integrating gene-centered data from ~ 150 web sources^53^. Gene annotation was performed for all 20,425 gene transcripts (out of 57,849) with log2fpkm ≥ 0.20 in platelets and/or megakaryocytes.

For all relevant transcripts of protein-coding genes (log2fpkm ≥ 0.20), a supervised classification procedure was developed to combine the corresponding proteins into function classes. The classification was hierarchical, according to a yes/no decision tree (Fig. 1), instructed by the EMBL UniProt-KB knowledgebase (visited January 2019–January 2020)^54^. UniProt-based decisions were based on the general description in Uniprot-KB of the (putative) protein's intracellular location and cellular function. Priority order of decision assignment was according to classical cell biology, *i.e.* from central' to 'peripheric: nucleus → mitochondria → endoplasmic reticulum and Golgi apparatus → cell → other cellular vesicles (lysosomes, peroxisomes, endosomes, secretory vesicles) → (plasma) membrane interactions → cytoskeleton structures → cytosolic protein types. When no relevant information was available, proteins were classified as 'Uncharacterized and other proteins'. Note that (assumed) extracellular proteins were classified as secretory proteins, as these are considered to be released into the blood plasma by gland cells.

### Area analysis of proteome-transcriptome space

For the matrix of 3,626 proteins with information on copy numbers and transcript levels in platelets (log2fpkm × 1000), a rectangular triangle was obtained, in which five areas (I-V) were pre-defined as follows. Top right corner, I (*x* = 100,000, *y* = 8, *x*-radius = 0.4, *n* = 58 PLT); top left corner, II (*x* = 1000, *y* = 8, *x*-radius = 0.3, n = 776 PLT), bottom left corner, III (*x* = 1000, *y* = 0.75, *x*-radius = 0.3, *n* = 137 PLT); middle of triangle, IV (*x* = 5000, *y* = 4, *x*-radius = 0.4, *n* = 928 PLT), and all below the triangle, V (*x* = 600–200,000, *y* = 0.6–10.2, *n* = 185 PLT). For each dot (protein) in the matrix, using Matlab the distance (in log space) was determined to each of the predefined areas; and recordings were made as in/out. Subsequently, for the proteins per function class, *p*-values of over-representation in pre-defined areas were calculated, employing a native Matlab function.

### Proteome prediction modelling

For prediction of the 'missing' (non-identified) part of the platelet proteome, we generated a model that was based on the definition, per protein class of three restraining factors: (i) low protein copy number, (ii) low mRNA level, and (iii) protein retainment in megakaryocytes upon proplatelet formation. Therefore, per function class, the fraction of non-identified proteins was calculated from all transcripts with log2fpkm ≥ 0.20 in platelets and/or megakaryocytes, with an arbitrary setting of well-identified classes having < 45% 'missing proteins'. Classes with low copy numbers were obtained from the proteome-transcriptome matrix (over-representation in areas II and III); or when no other explanation for low identification was present. Classes with low mRNA levels were also taken from the proteome-transcriptome space (over-representation in area V); or when the transcript fraction with log2fpkm 0.20–1.00 was > 22.5% (arbitrary set at half of 45%). Classes with supposed protein retainment in megakaryocytes came from handbook knowledge, i.e. the 'nuclear classes' C_13_ and C_20_; and furthermore C_3_-cytoskeleton microtubule, given the retainment of mitotic spindle and centromere structures. Mean restraining factors were calculated from the averages of non-identified proteins in the corresponding classes. See further Suppl. Methods. Coverage of hemostatic pathways was checked in the Reactome database^55^.

### Model validation using extended novel proteome

To validate our model, platelet samples were collected as above from 30 healthy subjects, digested with trypsin, and analyzed by liquid chromatography-mass spectrometry. See further Suppl. Methods. Mass spectrometry proteomics data were deposited to the ProteomeXchange Consortium via the PRIDE partner repository^56^ with the dataset identifier PXD022011 (username: reviewer_pxd022011@ebi.ac.uk; password: 7BeFQOxP).

### Bioinformatics and statistics

Statistical comparison was by probability analysis in Excel (Mann–Whitney U-test or Student t-test for continuous variables). Distribution profiles were compared by a χ^2^ test. Values of *p*<0.05 were considered significant.

## Supplementary Information

Supplementary Information 1. Datafile 1. Genome-wide transcriptome and identified proteome of PLT and MGK

Supplementary Information 2. Datafile 2. Identified proteins in cohorts 1-6

Supplementary Information 3. Datafile 3. Validation proteome and newly identified proteins

Supplementary Information 4. Datafile 4. Nodes of protein interaction network of T&H

Supplementary Information 5. Supplementary methods, figures and tables
