# Supplementary material for: Assessment of a complete and classified platelet proteome from genome-wide transcripts of human platelets and megakaryocytes covering platelet functions
Source: Sci Rep. 2021 Jun 11;11:12358. doi: 10.1038/s41598-021-91661-x (PMC8196183; doi:10.1038/s41598-021-91661-x)
Supplement: Supplementary file 5 — Supplementary Information 5. Supplementary methods, figures and tables [file 41598_2021_91661_MOESM5_ESM.pdf]

Supplementary file

Assessment of a complete and classified platelet proteome from genome-wide transcripts of human platelets and megakaryocytes covering platelet functions

Jingnan Huang, Frauke Swieringa,* Fiorella A. Solari,* Isabella Provenzale, Luigi Grassi, Ilaria De Simone, Constance C. F. M. J. Baaten, Rachel Cavill, Albert Sickmann,* Mattia Frontini,* Johan W. M. Heemskerk*

Index

Suppl. methods

References

Suppl. Figure 1. Buildup of identified human platelet proteome.

Suppl. Figure 2. Correlation of platelet and megakaryocyte transcriptomes.

Suppl. Figure 3. Comparison of relevant transcripts with(out) identified proteins.

Suppl. Figure 4. Distribution profile of transcripts per protein function class.

Suppl. Table 1. Clustering of proteins in quantitative proteome-transcriptome space.

Suppl. Table 2. Restraining factors and prediction model of full platelet proteome.

Supplementary datafile

Datafile 1 Genome-wide transcriptome and identified proteome of PLT and MGK.

Datafile 2. Identified proteins in cohorts 1-6.

Datafile 3. Validation proteome and newly identified proteins.

Datafile 4. Nodes of protein interaction network of T&H.

Supplementary Methods

Novel combined proteome analysis for validation. Well-purified platelet samples were obtained from 30 healthy subjects, and digested with trypsin using filter aided sample preparation, as described in the methods section. After digestion, 2 µg of peptide mixture from each sample were pooled. The pooled sample was desalted using a SpecVarian C_{18} cartridge, according to manufacturer instructions (Agilent, Santa Clara, California). 50 µg of the pooled sample was fractionated by high pH-

reversed phase chromatography (C_{18} column; BioBasic-18, 0.5 mm ID x 15 cm, 5 µm particle size, 300 Å pore size, (Thermo Scientific) using a linear gradient ranging from 5-38% of solvent B (mobile phase A: 10 mM ammonium formate, pH 8.0, B: 10 mM ammonium formate 84% acetonitrile, pH 8.0) for 90 minutes. Thirty fractions were collected every minute in a concatenated mode and dried under vacuum. Each fraction was analyzed on a Q Exactive HF mass spectrometer on line coupled to a U3000 RSLCnano (both from Thermo Scientific). Separate peptides fractions were loaded onto a trap column (Acclaim PepMap100 C_{18} trap column; 100 µm x 2 cm) with 0.1% trifluoroacetic acid at a flow rate of 20 µL/min, followed by separation of peptides on the main column (PepMap100 C18; 75 µm × 50 cm), using a non-linear gradient ranging from 7-24-38% of solvent B (84% acetonitrile, 0.1% formic acid) for 150 minutes. On the Q Exactive HF, a 90 min acquisition time was used, where survey scans were acquired at resolution of 30,000 using an automatic gain control (AGC) target value of 3 $\times 10^6$. MS/MS spectra of the top 15 most intense ions were acquired with a resolution of 15,000 an isolation width of 1.2 *m/z*, a normalized collision energy of 27%, an AGC target value of 5 $\times 10^4$ ions, a maximum injection time of 200 ms. Raw data were searched in Proteome Discoverer (Thermo Scientific) using Uniprot-KD (Human Uniprot 23/07/2018), with trypsin as an enzyme, carbamido-methylation as fixed modification, and oxidation of methionine as variable modification. For MS spectra, the mass tolerance was set to 10 ppm, and for MS/MS spectra, the mass tolerance was set to 0.02 Da. Obtained in the validation cohort were 5,505 unique proteins, which were separated into previously identified and newly identified, and were compared per corresponding gene with transcriptome data (Suppl. Datafile 2).

Technical limitations for obtaining the full platelet proteome. Integral membrane proteins (C_{10} and vesicular protein classes) with low relative abundance and high hydrophobicity are usually under-represented[1,2]. Use of specific enrichment steps can help here[3]. Second, while trypsin is the common choice as a digesting enzyme (cleaving at lysine and arginine residues), it is less efficient for domains rich in negatively charged amino acids[4]. This can be overcome by the use of other proteases or by changing digestion conditions. Third, loss of peptides is unavoidable in digested samples during sample preparation (FASP, precipitation, enrichment, desalting, elution)[5], and by the choice of protein denaturation agent Proc, 2010 #480}. Fourth, data-dependent acquisition (DDA) is the most common procedure to obtain mass

spectrometric data, however due to the TopN method used in DDA experiments, some precursors could be under- represented due to the defined threshold. Alternatively, in the data-independent acquisition (DIA) all precursors detected within a defined mass window are selected for MS/MS fragmentation and acquisition. This window is stepped across the entire mass range to collect MS/MS data from all detected precursors[6]. Furthermore, partial post-translational modifications (N-terminal acetylation, serine/threonine phosphorylation) can complicate the data analysis[7,8]. Concerning mass spectra analysis, current algorithms search for peptide-spectrum matches with 1% false discovery rate, estimated by a target-decoy search strategy[9-11], but this threshold may be less reliable for large data sets[12,13]. From our own data, we calculated that (with the exception of classes C_{02}, C_{17}, C_{21}).

References

1. Vuckovic, D., Dagley, L.F., Purcell, A.W. & Emili, A. Membrane proteomics by high performance liquid chromatography-tandem mass spectrometry: analytical approaches and challenges. *Proteomics* **13**, 404-423 (2013).
2. Vit, O. & Petrak, J. Integral membrane proteins in proteomics. How to break open the black box? *J. Proteomics* **153**, 8-20 (2017).
3. Lewandrowski, U. *et al.* Platelet membrane proteomics: a novel repository for functional research. *Blood* **114**, e10-e19 (2009).
4. Giansanti, P., Tsiatsiani, L., Low, T.Y. & Heck, A.J. Six alternative proteases for mass spectrometry-based proteomics beyond trypsin. *Nat. Protocols* **11**, 993-1006 (2016).
5. Mendes Maia, T. *et al.* Simple peptide quantification approach for MS-based proteomics quality control. *ACS Omega* **17**, 6754-6762 (2020).
6. Kalli, A., Smith, G.T., Sweredoski, M.J. & Hess, S. Evaluation and optimization of mass spectrometric settings during data-dependent acquisition mode: focus on LTQ-Orbitrap mass analyzers. *J. Proteome Res.* **12**, 3071-3086 (2013).
7. Zhang, X., Ye, J., Engholm-Keller, K. & Hojrup, P. A proteome-scale study on in vivo protein Nα-acetylation using an optimized method. *Proteomics* **11**, 81-93 (2011).
8. Hu, A., Noble, W.S. & Wolf-Yadlin, A. Technical advances in proteomics: new developments in data-independent acquisition. *F1000 Res.* **4**, 419 (2016).

9. Reiter, L. *et al.* Protein identification false discovery rates for very large proteomics data sets generated by tandem mass spectrometry. *Mol. Cell. Proteomics* **8**, 2405-2417 (2009).
10. Elias, J.E. & Gygi, S.P. Target-decoy search strategy for increased confidence in large-scale protein identifications by mass spectrometry. *Nat. Methods* **4**, 207-214 (2007).
11. Jeong, K., Kim, S. & Bandeira, N. False discovery rates in spectral identification. *BMC Bioinformatics* **13**, Suppl. 16, S12 (2012).
12. Savitski, M.M., Wilhelm, M., Hahne, H., Kuster, B. & Bantscheff, M. A scalable approach for protein false discovery rate estimation in large proteomic data sets. *Mol. Cell. Proteomics* **14**, 2394-2404 (2015).
13. Zhang, Y. *et al.* ProteinInferencer: confident protein identification and multiple experiment comparison for large scale proteomics projects. *J. Proteomics* **129**, 25-32 (2015).

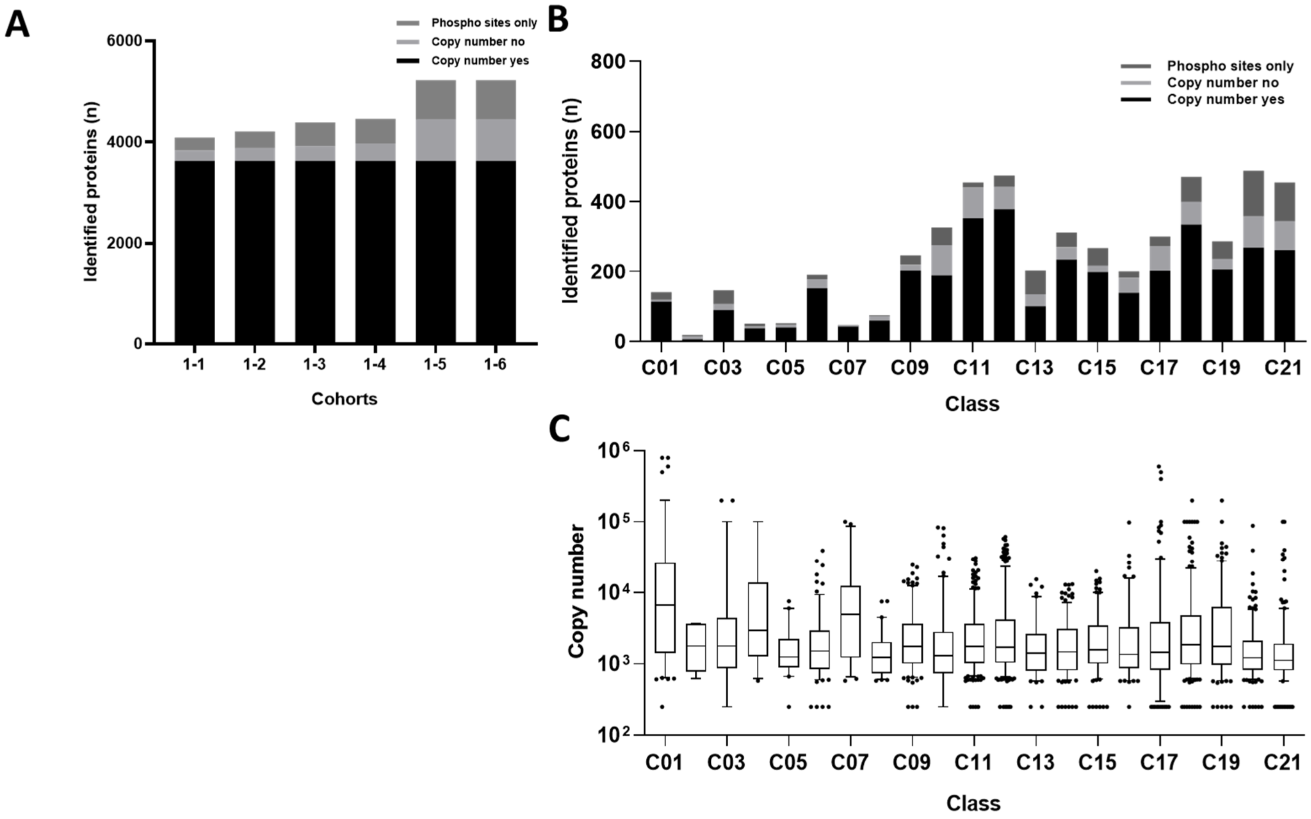

Suppl. Figure 1. *Buildup of identified human platelet proteome.* **A**, Progressive buildup of platelet proteome, obtained in six cohort studies (numbered 1 to 6) with healthy subjects. Black = 3,629 proteins with copy numbers; light gray = additional protein without copy numbers; dark gray = additional proteins by TiO_2 enrichment of phosphoproteome. **B**, Assignment of 5,211 identified proteins (all 6 cohorts) to 21 function classes (see Fig. 1). **C**, Ranges of copy numbers per function class.

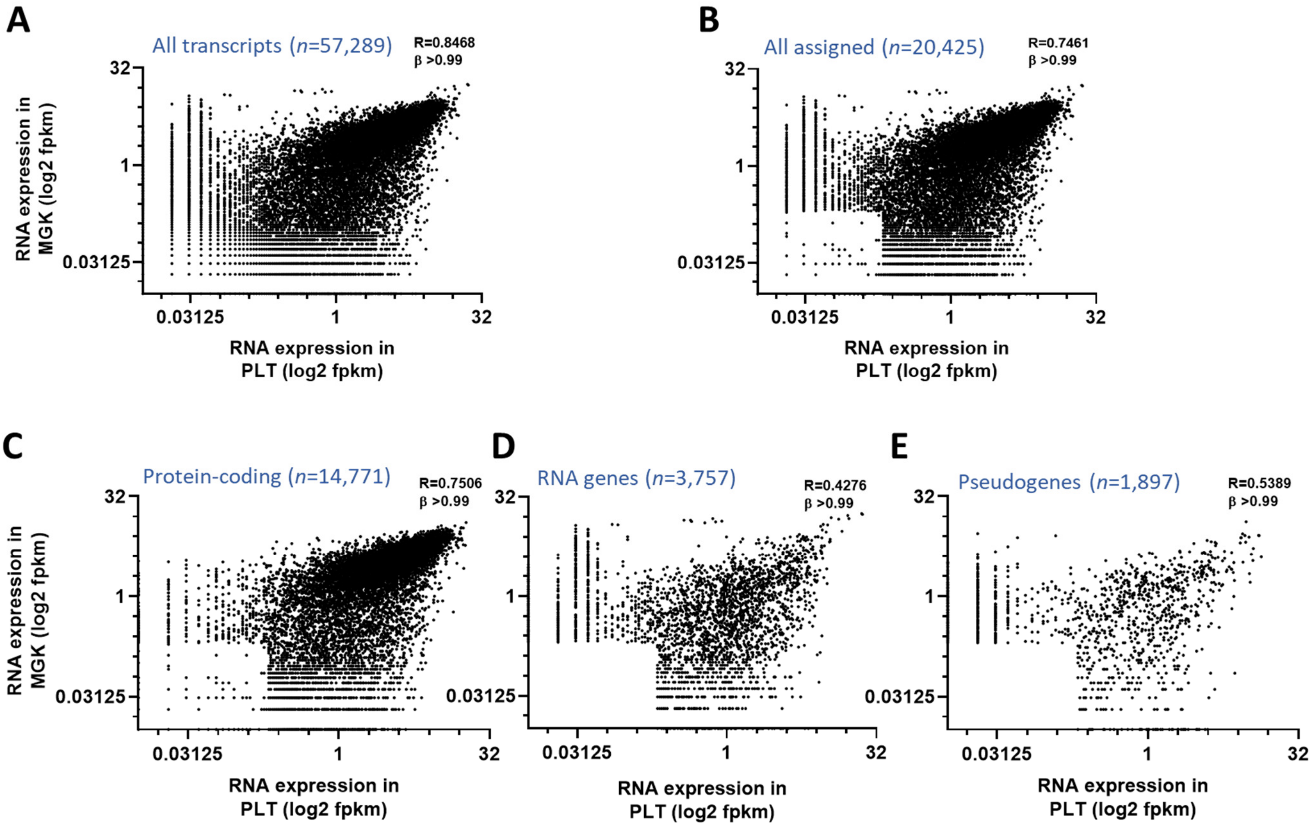

Suppl. Figure 2. *Correlation of platelet (PLT) and megakaryocyte (MGK) transcriptomes.* **A**, Correlation of all transcript levels in PLT *vs.* MGK genome-wide. **B**, Correlation of relevant transcripts (log2fpkm ≥0.20) in PLT and MGK. **C-E**, Correlation of relevant transcripts separated out between protein-coding genes (**C**), RNA genes (**D**) and pseudogenes (**E**).

A Transcripts with proteins

PLT and/or MGK	total >0.20	yes >0.20	no >0.20	yes <0.20
C01 Cytoskeleton actin-myosin	237	132	105	9
C02 Cytoskeleton intermediate	27	8	19	11
C03 Cytoskeleton microtubule	420	140	280	6
C04 Cytoskeleton receptor-linked	69	51	18	0
C05 Endosome proteins	101	52	49	0
C06 ER & Golgi proteins	568	190	378	1
C07 Glucose metabolism	58	46	12	1
C08 Lysosome & peroxisome proteins	168	74	94	1
C09 Membrane & protein trafficking	349	243	106	4
C10 Membrane receptors & channels	1430	318	1112	9
C11 Mitochondrial proteins	814	454	360	1
C12 Other metabolism	877	469	408	6
C13 Other nuclear proteins	1469	200	1269	3
C14 Proteasomal proteins	677	311	366	1
C15 Protein kinases & phosphatases	481	266	215	2
C16 Protein processing	308	199	109	1
C17 Secretory proteins	811	228	583	73
C18 Signaling & adapter proteins	1024	463	561	8
C19 Small GTPases & regulators	483	284	199	3
C20 Transcription & translation	2280	485	1795	3
C21 Uncharacterized & other proteins	2120	437	1683	18
Protein coding	14771	5050	9721	161
RNA genes	3757	0	3757	0
Pseudogenes	1897	0	1897	0
**Total transcripts**	**20425**	5050	15375	161

B Transcripts with proteins

PLT	total >0.20	yes >0.20	no >0.20	yes <0.20
C01	230	132	98	9
C02	26	8	18	11
C03	416	139	277	7
C04	69	51	18	0
C05	98	52	46	0
C06	549	190	359	1
C07	58	46	12	1
C08	164	74	90	1
C09	344	242	102	5
C10	1344	315	1029	12
C11	805	454	351	1
C12	855	467	388	8
C13	1442	200	1242	3
C14	665	310	355	2
C15	477	266	211	2
C16	301	197	104	3
C17	756	223	533	78
C18	1008	462	546	9
C19	478	284	194	3
C20	2230	484	1746	4
C21	1982	434	1548	21
Protein coding	14297	5030	9267	181
RNA genes	2480	0	2480	0
Pseudogenes	852	0	852	0
Total transcripts	**17629**	5030	12599	181

C Transcripts with proteins

MGK	total >0.20	yes >0.20	no >0.20	yes <0.20
C01	186	125	61	16
C02	20	6	14	13
C03	355	133	222	13
C04	64	48	16	3
C05	88	52	36	0
C06	478	190	288	1
C07	52	45	7	2
C08	152	74	78	1
C09	313	240	73	7
C10	914	300	614	27
C11	783	454	329	1
C12	781	463	318	12
C13	1378	196	1182	7
C14	638	311	327	1
C15	432	264	168	4
C16	289	198	91	2
C17	429	155	274	146
C18	829	446	383	25
C19	432	281	151	6
C20	2111	482	1629	6
C21	1772	419	1353	36
Protein coding	12496	4882	7614	329
RNA genes	2783	0	2783	0
Pseudogenes	1564	0	1564	0
Total transcripts	**16843**	4882	11961	329

Suppl. Figure 3. *Comparison of relevant transcripts with or without identified platelet proteins.* **A, C.** Listing per function class of numbers of relevant transcripts (log2fpkm ≥0.20) that were yes/no identified in the platelet proteome. Transcriptomes were combined from platelets (PLT) and megakaryocytes (MGK), or used from PLT or MGK only. Indicated in gray are proteins without relevant mRNA expression (log2fpkm <0.20). Transcripts were summed as protein coding, RNA genes and pseudogenes. Data are shown for: **A**, Combined PLT/MGK transcriptome; **B**, PLT transcriptome; **C**, MGK transcriptome.

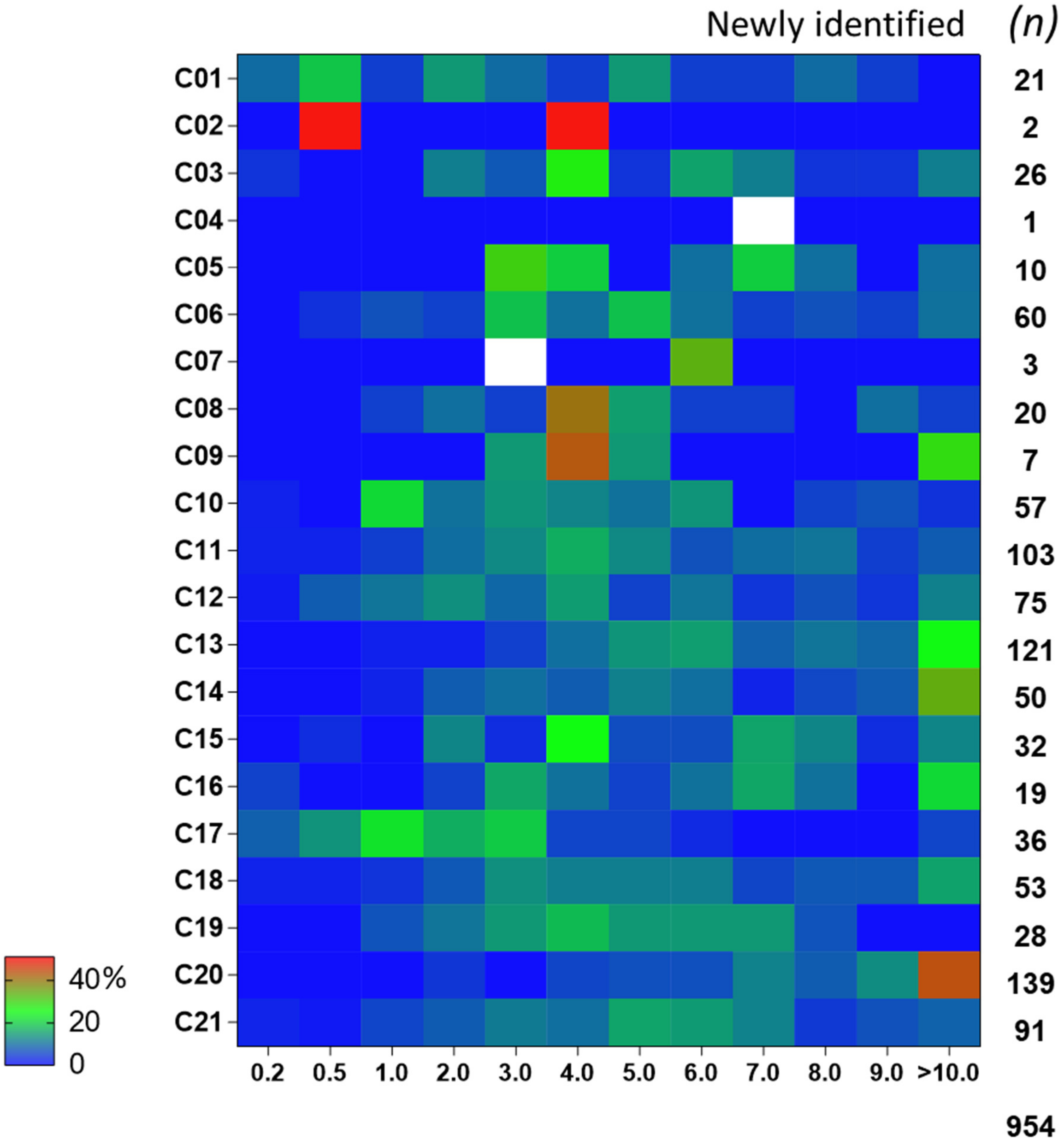

Suppl. Figure 4. *Distribution profile of transcripts of newly identified proteins per protein function class.* Heatmap of percentual distribution of transcript levels per function class (rainbow colors; blue = low, red = high) for 954 newly identified proteins in validation cohort. Numbers per class are indicated in right column.

Suppl. Table 1. *Clustering of proteins per class in quantitative proteome-transcriptome space. P*-values are given, representing significance of over-representation of protein in defined areas I-V of proteome-transcriptome matrix, based on transcript levels in platelets (PLT) or megakaryocytes (MGK). For visualization of areas I-V, see Fig. 4C-D. Modelling was as described in the methods section. Statistical significance increasing with red coloring.

Protein function class	High transl. high transcr.		High transl. low transcr.		Low trans. low transcr.		Mid transl. mid transcr.		Unrelevant transl.		Protein count		
	Area I		Area II		Area III		Area IV		Area V		Area I-V		
P-value (Matlab)	PLT	MGK	PLT	MGK	PLT	MGK	PLT	MGK	PLT	MGK	PLT	MGK	Total
01 Cytoskeleton actin-myosin	1.31E-09	1.10E-10	9.92E-01	9.99E-01	6.32E-01	8.82E-02	9.92E-01	9.74E-01	1.71E-03	4.22E-04	66	66	114
02 Cytoskeleton intermediate	1.00E+00	1.00E+00	8.15E-01	1.00E+00	1.00E+00	9.98E-02	1.00E+00	9.13E-01	3.93E-03	2.76E-04	4	6	7
03 Cytoskeleton microtubule	5.27E-04	3.00E-03	5.93E-01	4.17E-01	6.76E-01	7.49E-01	5.69E-01	5.24E-01	9.06E-02	1.27E-01	60	56	91
04 Cytoskeleton receptor-linked	2.90E-03	2.90E-03	7.36E-01	9.97E-01	7.70E-01	4.36E-01	6.67E-01	8.33E-01	8.65E-01	8.86E-01	22	16	38
05 Endosome proteins	1.00E+00	1.00E+00	1.30E-01	1.99E-01	7.88E-01	1.00E+00	7.30E-01	7.85E-01	8.78E-01	1.00E+00	23	18	40
06 ER & Golgi proteins	1.00E+00	1.00E+00	1.17E-01	3.41E-02	3.51E-01	6.69E-01	6.00E-01	7.24E-01	1.00E+00	1.00E+00	85	74	152
07 Glucose metabolism	4.57E-03	4.57E-03	9.69E-01	9.56E-01	7.73E-02	1.00E+00	2.93E-01	1.01E-01	6.53E-01	9.14E-01	28	25	43
08 Lysosome & peroxisome proteins	1.00E+00	1.00E+00	1.98E-01	6.34E-01	3.97E-01	1.00E+00	9.65E-01	9.34E-01	9.58E-01	9.68E-01	30	22	60
09 Membrane & protein trafficking	1.00E+00	1.00E+00	4.10E-01	8.06E-01	9.85E-01	8.13E-01	3.27E-03	9.77E-03	9.81E-01	9.99E-01	122	105	202
10 Membrane receptors & channels	2.20E-01	2.20E-01	1.03E-01	3.73E-02	9.53E-01	3.55E-01	1.00E+00	9.93E-01	5.88E-01	5.65E-01	98	103	202
11 Mitochondrial proteins	1.00E+00	1.00E+00	1.00E+00	1.00E+00	5.49E-04	4.31E-01	1.31E-04	1.24E-04	1.00E+00	1.00E+00	187	170	353
12 Other metabolism	7.40E-01	8.71E-01	1.00E+00	9.64E-01	1.19E-01	6.80E-01	2.82E-02	4.05E-02	9.96E-01	1.00E+00	203	186	379
13 Other nuclear proteins	1.00E+00	1.00E+00	6.03E-01	4.69E-01	7.43E-01	1.00E+00	6.16E-01	7.64E-01	9.69E-01	9.25E-01	51	45	101
14 Proteasomal proteins	1.00E+00	1.00E+00	3.58E-01	1.84E-02	9.82E-01	6.90E-01	6.62E-03	1.32E-02	1.00E+00	1.00E+00	136	134	235
15 Protein kinases & phosphatases	1.00E+00	1.00E+00	1.77E-02	4.60E-01	9.83E-01	5.73E-01	1.74E-02	3.55E-03	9.93E-01	9.96E-01	126	112	198
16 Protein processing	8.98E-01	8.98E-01	8.16E-01	3.14E-02	7.78E-02	6.20E-01	3.47E-01	7.43E-01	5.71E-01	1.00E+00	81	70	139
17 Secretory proteins	8.43E-01	8.43E-01	1.00E+00	1.00E+00	8.38E-03	2.78E-05	1.00E+00	1.00E+00	1.43E-52	2.78E-18	141	155	202
18 Signaling & adapter proteins	8.09E-02	3.67E-02	3.70E-02	8.74E-01	9.90E-01	7.47E-01	2.53E-01	2.28E-02	1.00E+00	2.53E-81	196	177	334
19 Small GTPases & regulators	2.34E-01	2.34E-01	2.82E-02	5.65E-01	1.00E+00	1.00E+00	3.97E-01	3.46E-01	9.95E-01	1.00E+00	121	102	207
20 Transcription & translation	9.89E-01	9.89E-01	2.78E-08	3.35E-11	9.98E-01	1.00E+00	9.95E-01	9.96E-01	9.99E-01	9.98E-01	156	146	268
21 Uncharacterized & other proteins	9.87E-01	9.87E-01	2.64E-02	4.61E-01	8.46E-03	5.52E-01	1.00E+00	1.00E+00	1.75E-01	9.98E-01	148	108	261
All classes											2084	1896	3626

Suppl. Table 2. *Restraining factors per function class and prediction model of full platelet proteome.* Analysis of non-identified proteins (*n*=9,721) from the relevant, combined PLT/MGK transcriptome per function class. Indicated in **blue** are fractions transcripts present in the identified proteome, and fractions with low mRNA (arbitrarily set at log2fpkm <1.00). Indicated in **green** (ID) are well-identified classes with fractions >0.55 identified. Indicated in **red** is indication of one or more restraining factors per class: *(i)* over-representation of low copy number (areas II-III in Fig. 4D), *(ii)* low mRNA level (area V, LM = low mRNA >45%); *(iii)* retainment in megakaryocyte (peri)nucleus upon platelet shedding (RET).

**Protein function class** (n)	**Fraction not identified**		**Fraction low mRNA**		**Mostly identified**	**Explanation for low identification**		
UniProt-KB: in protein name (n≥10)	(fraction)		(fraction)		>55%	**Low copy number** (II, III)	**Low mRNA** (>20%, V)	**Retained in MGK**
**C01 Cytoskeleton actin-myosin** (237)	0.44		0.30		ID		LM, V	
Actin (36)		0.14		0.40				
Myosin (45)		0.44		0.55				
**C02 Cytoskeleton intermediate** (27)	0.70		0.42		NID		LM, V	
Keratin (12)		0.58		0.57				
**C03 Cytoskeleton microtubule** (420)	0.67		0.19		NID			RET
Centromere (16)		0.88		0.21				
Centrosomal (36)		0.75		0.11				
Dynein (41)		0.61		0.40				
Kinesin (38)		0.71		0.37				
Mitotic spindle / HAUS (13)		0.69		0.22				
Tubulin (74)		0.46		0.29				
**C04 Cytoskeleton receptor-linked** (69)	0.26		0.17		ID			
LIM / Wiskott (10)		0.30		0.33				
**C05 Endosome proteins** (101)	0.49		0.18		NID	?		
Multivesicular body (11)		0.18		0.00				
WAS / WASH (11)		0.45		0.00				
**C06 ER & Golgi proteins** (568)	0.67		0.24		NID		LM	
AP-1/3 complex subunit (19)		0.21		0.25				
ER membrane / lumen protein (34)		0.41		0.36				
Golgi (59)		0.47		0.57				
Trafficking protein particle (16)		0.25		0.00				
Transferase (95)		0.96		0.45				
**C07 Glucose metabolism** (58)	0.21		0.25		ID		LM	
Glucose (18)		0.33		0.17				
Fructose (11)		0.35		0.25				
**C08 Lysosome & peroxisome proteins** (168)	0.56		0.13		NID		LM	
Lysosome /lysosomal (21)		0.52		0.09				
Peroxisome / peroxisomal (37)		0.54		0.30				
V-type proton ATPase (17)		0.29		0.40				
**C09 Membrane & protein trafficking** (349)	0.30		0.31		ID		LM	
Exocyst complex (10)		0.10		1.00				
Protein transport (11)		0.09		0.00				
Sorting nexin (27)		0.37		0.40				
Synapto (19)		0.74		0.57				
Syntaxin (20)		0.20		0.00				
Vacuolar protein sorting-associated (26)		-		-				

Protein function class (n)	Fraction not identified	Fraction low mRNA	Mostly identified	Explanation for low identification		
UniProt-KB: in protein name (n≥10)	(fraction)	(fraction)	>55%	Low copy number (II, III)	Low mRNA (>20%, V)	Retained in MGK
**C10 Membrane receptors & channels** (1430)	0.78	0.39	NID		LM	
Calcium / Cal (42)	0.79	0.55				
Chemokine/Interleukin receptor (18)	0.83	0.60				
C-type lectin domain family (19)	0.95	0.56				
Glycoprotein (32)	0.75	0.38				
G-protein coupled (45)	0.98	0.68				
Integrin (30)	0.57	0.59				
Olfactory receptor (28)	1.00	0.86				
Purinoceptor (14)	0.79	0.36				
Solute carrier family / SLC (146)	0.64	0.46				
Voltage-dependent/gated (38)	0.84	0.63				
**C11 Mitochondrial proteins** (814)	0.44	0.10	ID	III		
ATP synthase (23)	0.26	0.17				
Cytochrome b/c (49)	0.37	0.22				
Import (27)	0.22	0.00				
NADH dehydrogenase (39)	0.18	0.29				
Ribosomal protein (84)	0.44	0.00				
tRNA (31)	0.39	0.08				
**C12 Other metabolism** (877)	0.47	0.22	NID		LM	
(Metabolite) kinase (69)	0.36	0.40				
(Metabolite) phosphatase (53)	0.43	0.35				
(Metabolite) reductase (49)	0.37	0.44				
(Metabolite) synthase (57)	0.49	0.29				
(Metabolite) transferase (162)	0.53	0.43				
**C13 Other nuclear proteins** (1469)	0.86	0.07	NID			RET
Chromatin (17)	0.76	0.08				
Histone (150)	0.89	0.10				
Nuclear pore complex (34)	0.82	0.32				
Polymerase (28)	0.86	0.00				
Repair protein (29)	0.86	0.12				
**C14 Proteasomal proteins** (677)	0.54	0.11	NID	II		
COP9 signalosome (10)	0.10	0.00				
E3 ubiquitin-protein ligase (180)	0.69	0.17				
Kelch-like (20)	1.00	0.30				
NEDD (15)	0.20	0.33				
Proteasome/ proteasomal (45)	0.18	0.13				
Ubiquitin-conjugating (32)	0.53	0.12				
**C15 Protein kinases & phosphatases** (481)	0.45	0.20	NID	II	LM	
Mitogen-activated protein kinase (41)	0.44	0.33				
Serine/threonine-protein kinase (144)	0.42	0.34				
Serine/threonine-protein phosphatase (40)	0.38	0.27				
Tyrosine (-protein) phosphatase (29)	0.45	0.54				
Tyrosine-protein kinase (29)	0.34	0.30				

Protein function class (n)	Fraction not identified	Fraction low mRNA	Mostly identified	Explanation for low identification		
UniProt-KB: in protein name (n≥10)	(fraction)	(fraction)	>55%	Low copy number (II, III)	Low mRNA (>20%, V)	Retained in MGK
**C16 Protein processing** (308)	0.35	0.13	ID			
Dol / dolichol (16)	0.00	-				
Glucosyl / glycosyltransferase (17)	0.18	0.00				
Methyltransferase (13)	0.62	0.00				
Palmitoyl / sialyl (20)	0.95	0.26				
Pept/ peptidyl (50)	0.26	0.31				
**C17 Secretory proteins** (811)	0.72	0.47	NID	III	LM, V	
Collagen (33)	0.73	0.71				
Growth factor (36)	0.61	0.64				
Interleukin / chemokine (41)	0.83	0.53				
Metalloproteinase (28)	0.82	0.61				
Protease (21)	0.81	0.59				
**C18 Signaling & adapter proteins** (1024)	0.55	0.26	NID	II	LM	
14-3-3 / S100 protein (15)	0.27	0.50				
Adapter / adaptor protein (35)	0.63	0.41				
Bcl / Bax (18)	0.50	0.44				
Calcium / calpain (38)	0.61	0.52				
cAMP / A-kinase (34)	0.76	0.46				
Caspase (19)	0.42	0.13				
cGMP / G kinase (17)	0.59	0.30				
Guanine nucleotide / G-protein (43)	0.42	0.67				
Phosphatidyl /phosphoinositide (66)	0.38	0.20				
**C19 Small GTPases & regulators** (483)	0.41	0.20	ID	II	LM	
Arf-GAP (18)	0.61	0.27				
Rab (78)	0.19	0.27				
Ral (12)	0.33	0.50				
Rap (17)	0.47	0.13				
Ras *(non RAb, Ral, Rap)* (20)	0.70	0.50				
Rho (88)	0.57	0.44				
**C20 Transcription & translation** (2280)	0.79	0.09	NID	II		RET
Nuclear (127)	0.67	0.04				
Ribosomal protein (96)	0.65	0.03				
Transcription (303)	0.87	0.82				
Translation (56)	0.21	0.08				
tRNA (71)	0.56	0.23				
Zinc finger (579)	0.98	0.28				
**C21 Uncharacterized & other proteins** (2120)	0.79	0.23	NID	III	LM	
Coiled-coil (91)	0.84	0.28				
FAM (255)	0.77	0.40				
Leucine-rich repeat (36)	0.83	0.60				
Putative (90)	0.89	0.39				
Transmembrane (133)	0.70	0.40				
Uncharacterized (249)	0.92	0.48				
